# Supplementary material for: An improved SNAP-ADAR tool enables efficient RNA base editing to interfere with post-translational protein modification
Source: Nat Commun. 2024 Aug 5;15:6615. doi: 10.1038/s41467-024-50395-w (PMC11300799; doi:10.1038/s41467-024-50395-w)
Supplement: Supplementary file 1 — Supplementary Information [file 41467_2024_50395_MOESM1_ESM.pdf]

An Improved SNAP-ADAR Tool Enables Efficient RNA Base Editing to Interfere with Post-translational Protein Modification

Karthika Devi Kiran Kumar,[1] Shubhangi Singh,[1] Stella Maria Schmelzle,[1] Paul Vogel,[2] Carolin Fruhner,[1] Alfred Hanswillemenke,[1] Adrian Brun,[1] Jacqueline Wettengel,[1] Yvonne Füll,[1] Lukas Funk,[1] Valentin Mast,[1] J. Josephine Botsch,[1] Philipp Reautschnig,[1] Jin Billy Li[2] and Thorsten Stafforst[1,3]

[1]Interfaculty Institute of Biochemistry, University of Tübingen (Germany)

[2]Department of Genetics, Stanford University, Stanford, CA (USA)

[3]Gene and RNA Therapy Center (GRTC), Faculty of Medicine University Tübingen (Germany)

<u>**Supplementary Figures**</u>

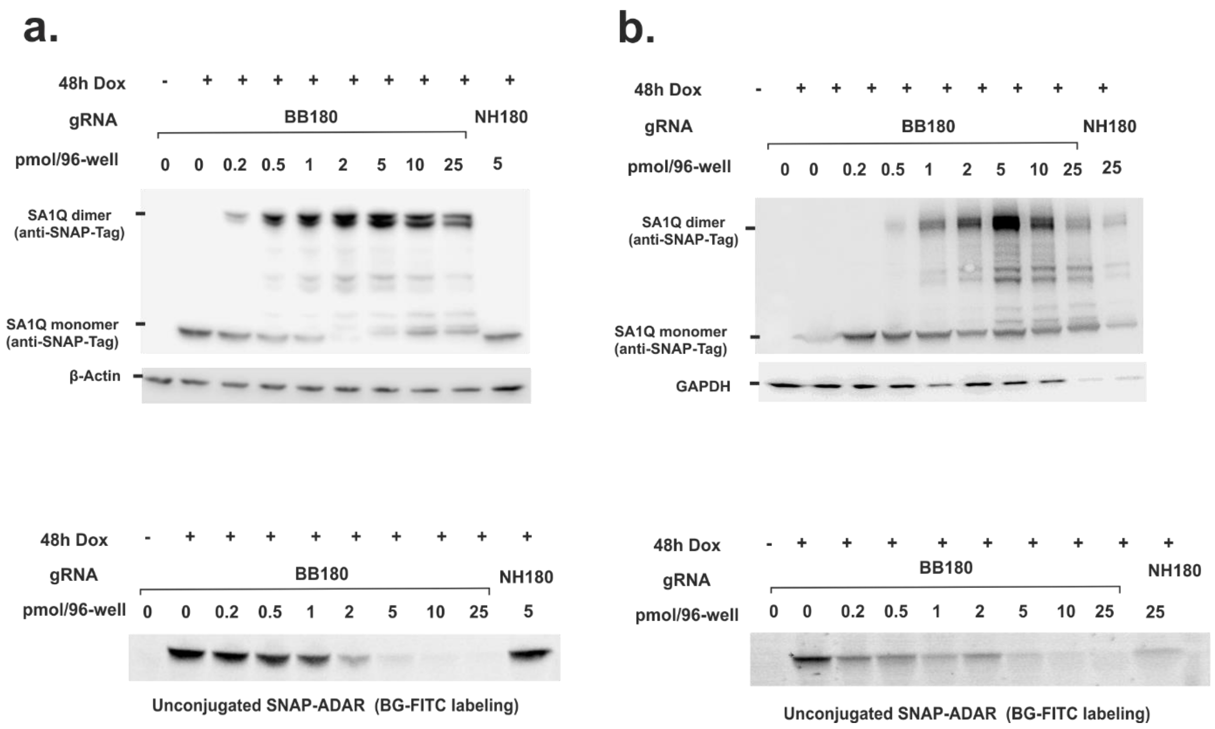

Supplementary Figure 1 Blots showing guideRNA dose-dependent dimerization of the effector SA1Q in Flp-In T-Rex 293 – SA1Q cells induced with 10ng/ml Doxycycline for 48 hrs. Cells were transfected with indicated amounts of BB180 (BisBG-25 nt + LNA gRNA against STAT1 Y701 site) or NH180 (gRNA lacking the BisBG moiety for SA attachment). **A)** and **B)** are biological replicates (N=2) of Fig.1d, with Western blot (blot on top) and BG-FITC -protein/gRNA conjugation assay blot (blot in the bottom) respectively, showing gRNA dose-dependent of SA1Q dimerization in cells induced for 48hrs with Doxycycline. GAPDH/ß-Actin served as loading controls. All guide RNA amounts denote pmol/96-well with 150 µl total volume. Western blotting and BG-FITC staining were done as described in the methods section. Full blots are available in the Source Data file.

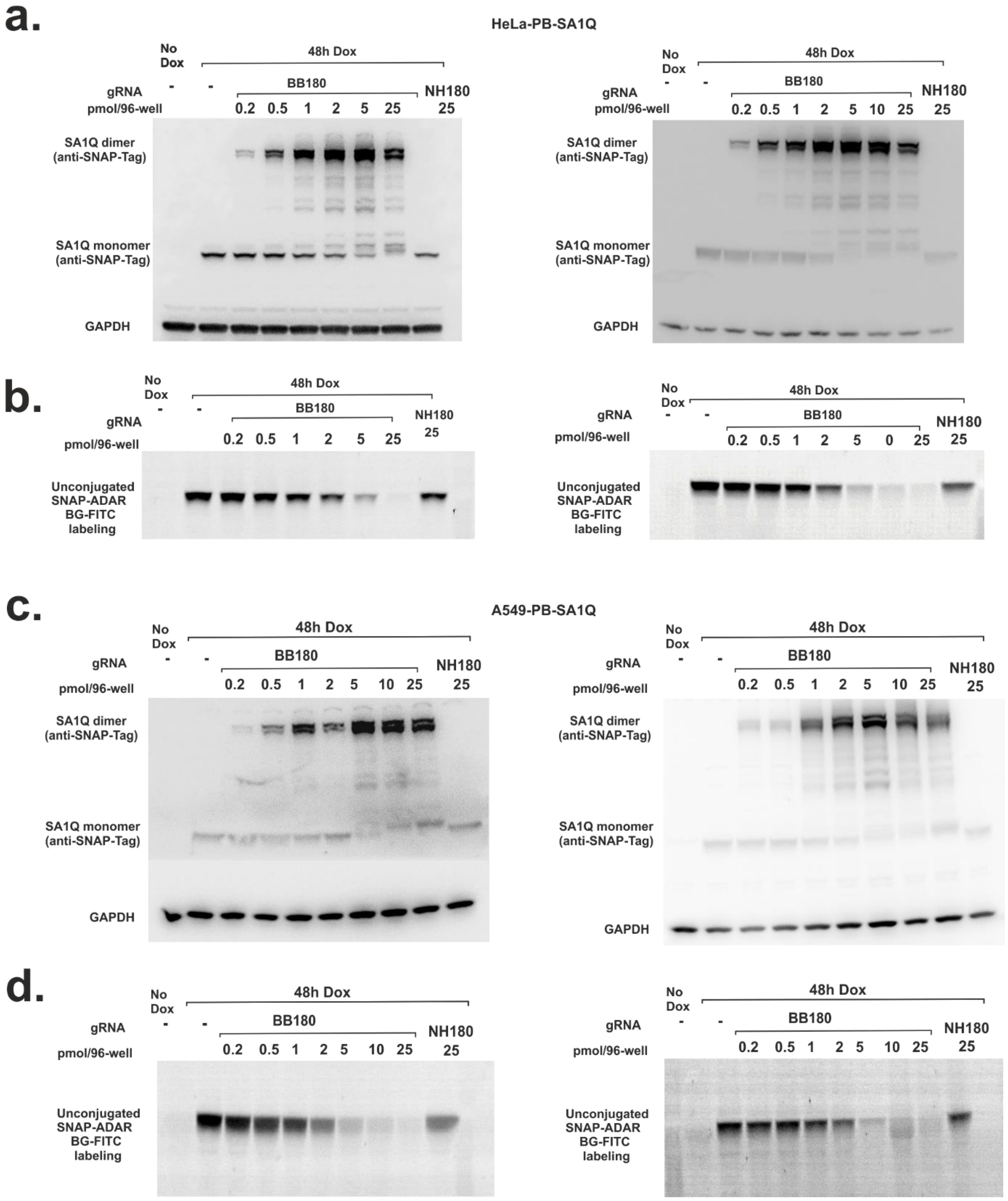

Supplementary Figure 2. Blots showing biological replicates (N=2) of SNAP-ADAR protein expression and its conjugation with gRNA studied in Hela-PB-SA1Q and A549-PB-SA1Q cells using Western blot and BG-FITC -protein/gRNA conjugation assay. Cells were transfected with different amounts of BisBG-25 nt + LNA gRNA against STAT1 Y701 site (BB180) in 96-well plate set up with 1µg/ml doxycycline induction for 48h. NH180 denotes gRNA lacking the BisBG moiety for SA attachment. **A)** and **B)** show Western blots and BG-FITC blots (replicates) respectively for Hela-PB-SA1Q cells. **C)** and **D)** show Western blots and BG-FITC blots (replicates) respectively for A549-PB-SA1Q cells. GAPDH served as loading control. All guide RNA amounts denote pmol/96-well with 150 µl total volume. Western blotting and BG-FITC staining were done as described in the methods section. Full blots are available in the Source Data file.

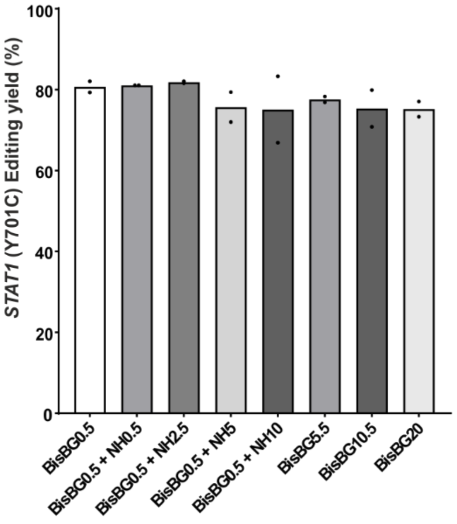

Supplementary Figure 3. Competitive editing experiment of editing-competent BisBG guide RNAs with editing-incompetent amino guide RNA of the same sequence and chemical modification. In Flp-In T-REx 293 SA1Q cells, the editing-competent BisBG 180 guide (0.5 pmol per 96 well) and an increasing amount (0.5-10 pmol per 96-well) of the same editing-incompetent NH180 control guide RNA against the transcript STAT1 (Y701>C site) were co-transfected. Even a 20x excess of amino control guide RNA (NH180) did not reduce the editing yield with 0.5 pmol BisBG guide RNA, indicating that the guide RNAs act on the target RNA rather in a hit-and-run fashion than staying at the target for a long time. Furthermore, editing yield for increasing BisBG guide RNA concentrations (up to 20 pmol/96 well) are shown. The data is presented as N=2 biological replicates, as indicated by individual data points. Source data are provided in Source Data file.

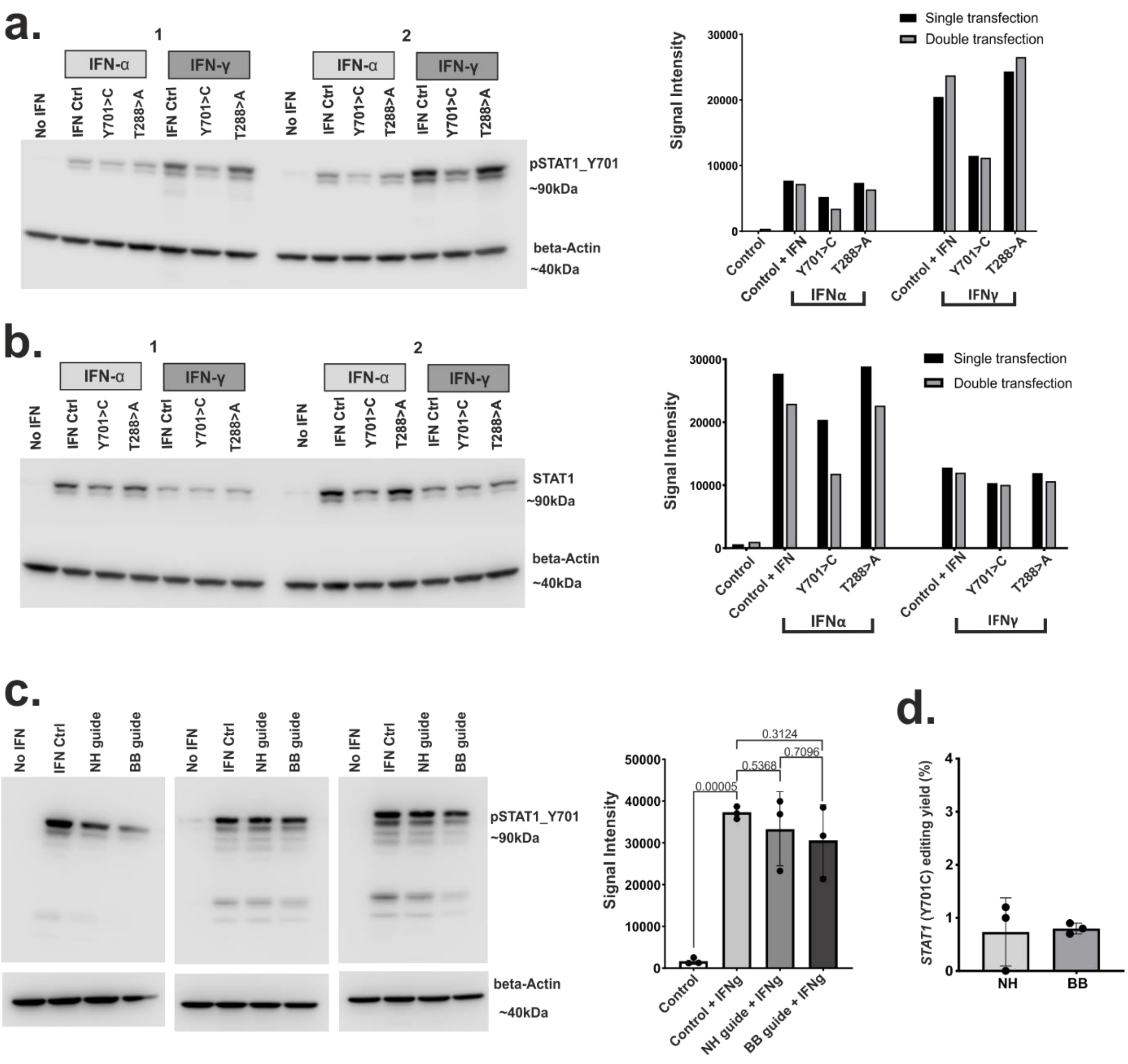

Supplementary Figure 4. Analysis of STAT1 activation (tyrosine 701 phosphorylation) by Western Blot in response to up (T288>A) and down regulating (Y701>C) PTMi. **A)** Shows phospho-STAT1 and **B)** shows total STAT1 in response to various PTMi settings and induction by interferon-α and -γ, as indicated. Experiment **1** in **A)** and **B):** Dox (1 µg/ml, 72h total), single transfection (T1) in 24 well plate with 2x10^5 cells and 8 pmol guide per well, 2000 u/ml IFN α or γ induction 24h prior to lysis. Experiment **2** in **A)** and **B):** Dox (1 µg/ml, 120h total), double transfection (T2) with 48h in between, in 24 well plate with $2x10^5$ cells and 8 pmol guide per well, 2000 U/ml IFN-α or -γ induction 24h prior to lysis. **C)** Effect of an editing-incompetent guide RNA that can (BB482 = BisBG 482) or cannot (NH482) recruit the SNAP-ADAR effector on target protein expression (pSTAT1) by Western Blot. The bar graph includes a statistical analysis of the band intensities, with P values indicated. Applied was a 2-winged t-test, N=3; P>0.05 is interpreted as non-significant. **D)** The editing-incompetent guide RNA BisBG 482 was unable to induce the Y701>C edit in STAT1. Source data are provided as a Source Data file.

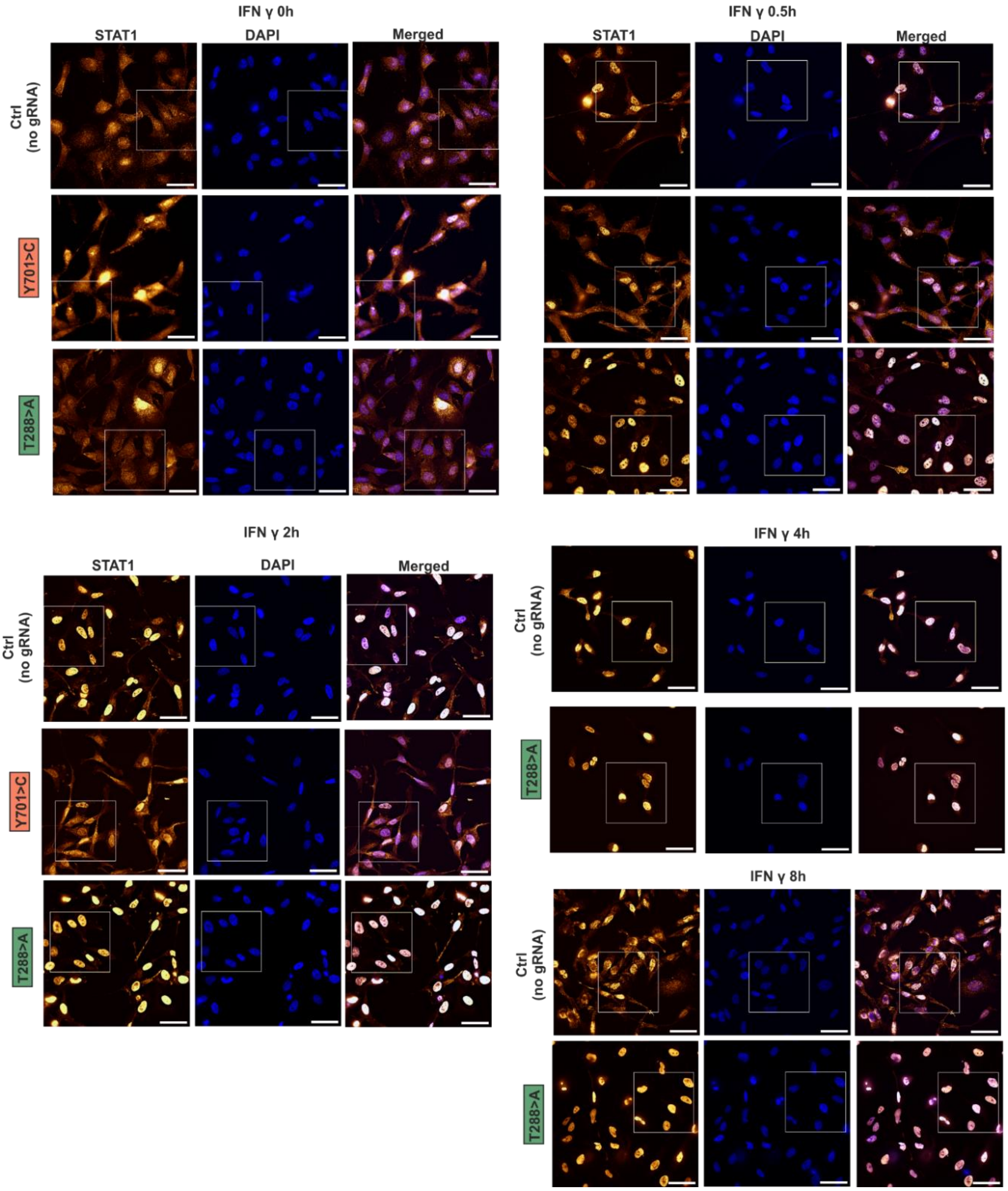

Supplementary Figure 5. Immunofluorescence imaging of total STAT1 cellular localization upon different gRNA treatment vs control (no gRNA), at various time points post IFN-γ induction. Insets show zoomed-in areas shown in Fig. 5d. For detailed protocol and list of antibodies, see the Methods section and Supplementary Table 1, respectively. Images were taken with a Nikon Eclipse Ti2-E inverted fluorescent microscope, equipped with a photometrics® Prime 95B camera and a lumencor® AuraII light engine. All pictures were recorded using a 60x oil objective (numerical aperture 1.4) and Olympus IMMOIL-F30CC immersion oil. The excitation wavelengths and corresponding filter sets used to record each channel are specified in Supplementary Table 2. The same acquisition settings were chosen for each channel for all images. The images were 3D deconvoluted, and further processed in FIJI ImageJ[1]. Amber LUT, used to display STAT1 protein, was obtained from ImageJ Wiki – NucMed LUT list [2].For more details on LUT, see Source Data.

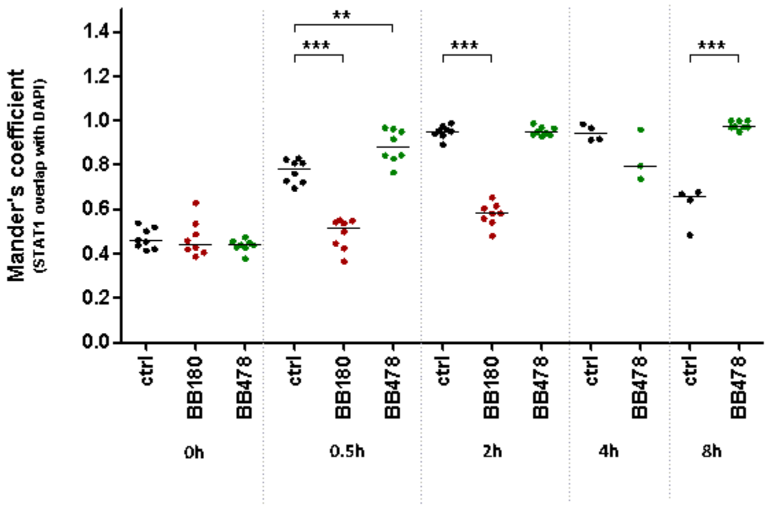

Supplementary Figure 6. Colocalization analysis. Mander's coefficient for the overlap of total STAT1 protein signal with nuclear DAPI signal was determined and plotted for each condition using the JACoP plug-in[3] in FIJI ImageJ[1]. An unbiased sectioning of biological replicate images (N =2 for most, N =1 for some) into quadrants was done. Significance for N= 3,4 or 8 sections was determined by an unpaired, two-tailed t-test, $P \leq 0.05$ = *, $P \leq 0.01$ = **, $P \leq 0.001$ = ***, $P>0.05$ = nonsignificant. Individual data points are given, the median is denoted by a black line. Source data are provided as a Source Data file.

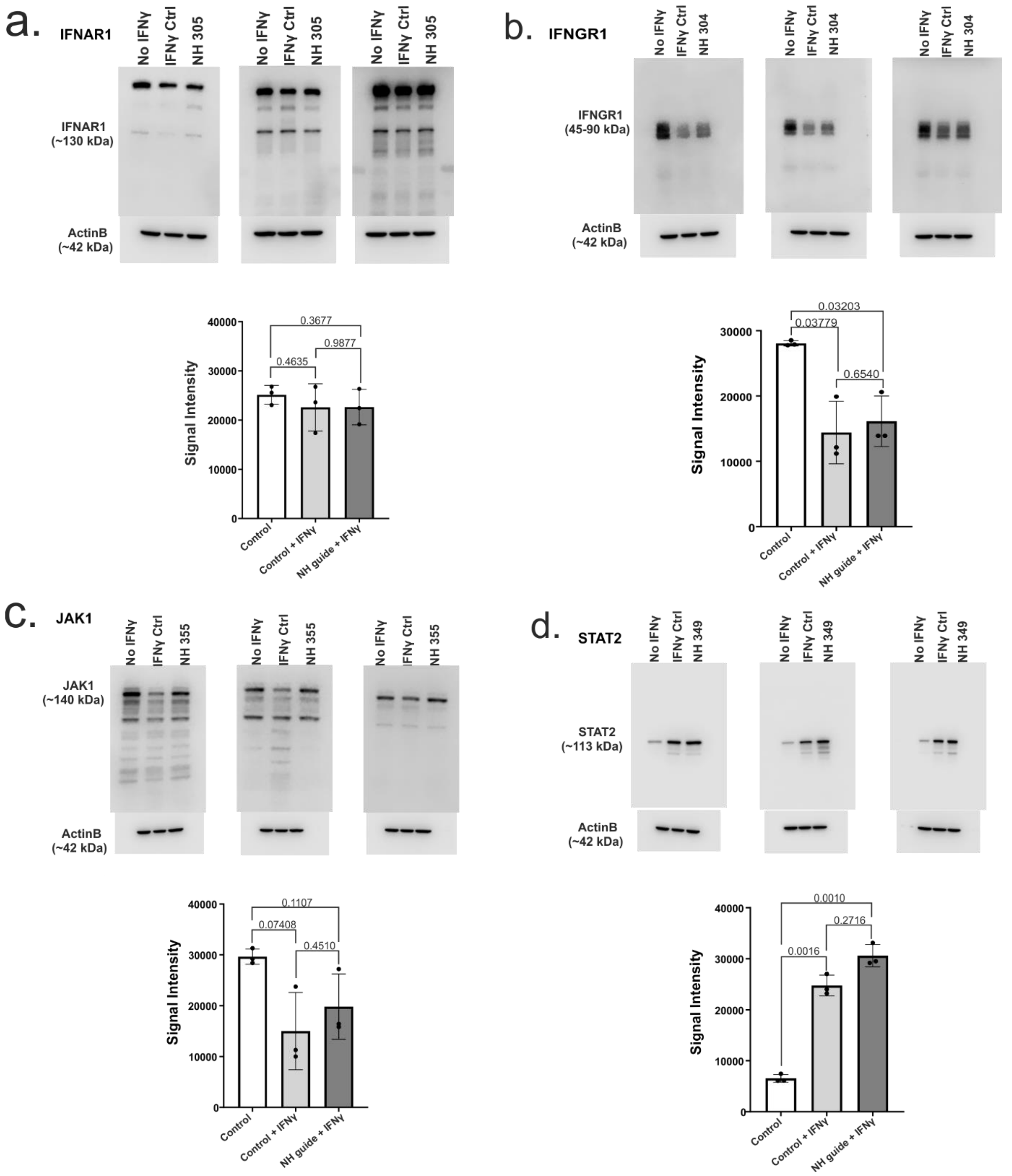

Supplementary Figure 7. Analysis of the effect of guide RNA transfection with or without IFN-γ induction on the protein expression of four different PTMi targets by Western Blot. The bar graphs show the statistical analysis of the band intensities, with P values indicated. Applied was a 2-winged t-test, N=3; P>0.05 is interpreted as non-significant. **A)** For the IFNAR1 target. **B)** For the IFNGR1 target. **C)** For the JAK1 target. **D)** For the STAT2 target. Dox (1 µg/ml, 120h total), double transfection (T2) with 48 h in between, in 24 well plate with $2x10^5$ cells and 8 pmol guide per well, 2000 u/ml IFN-γ induction 24 h prior to cell lysis. Full Western blots and Source data are provided as a Source Data file.

References

1. Schneider, C. A., Rasband, W. S. & Eliceiri, K. W. NIH Image to ImageJ: 25 years of image analysis. *Nat Methods* **9**, 671–675 (2012).
2. https://imagej.net/ij/download/luts/NucMed_Image_LUTs/.
3. BOLTE, S. & CORDELIÈRES, F. P. A guided tour into subcellular colocalization analysis in light microscopy. *J Microsc* **224**, 213–232 (2006).

Supplementary Tables

Supplementary Table 1 All antibodies were diluted in 5% dry milk in 1x TBST buffer.

**Antibody**	**Cat.no.**	**Manufacturer**	**Dilution**
Mouse α-Beta-Actin	A5441	Sigma	1: 5000
Rabbit α-SNAP-tag	P9310S	New England Biolabs	1: 1000
α-SNAP Tag Polyclonal Antibody	CAB4255	Thermo-Fisher Scientific	1: 1000
Mouse-α-GAPDH Loading Control Monoclonal Antibody (GA1R)	MA5-15738	Thermo-Fisher Scientific	1: 1000
Mouse-α-GAPDH Monoclonal Antibody (6C5)	AM4300	Thermo-Fisher Scientific	1: 1000
Rabbit-α-Gapdh (D16H11) XP	5174T/5174S	Cell Signalling	1: 2000
pSTAT1(Y701)	9167	Cell Signalling	1: 1000
STAT1 mouse	9176	Cell Signalling	1: 1000
STAT1 (42H3) Rabbit	9175	Cell Signalling	1: 1000
Goat α-Rabbit Alexa Fluor 594	8889S	Cell Signalling	1: 1000
Goat α-Rabbit HRP	111-035-003	Jackson Immuno Research Laboratories	1: 10000
Goat α-Mouse HRP	115-035-003	Jackson Immuno Research Laboratories	1: 10000
IFNAR1 rabbit	MA5-32006	Thermo-Fisher Scientific	1: 1000
JAK1 mouse	50996	Cell Signaling	1: 1000
STAT1 Rabbit	9172	Cell Signaling	1: 1000
IFNGR1 rabbit	34808	Cell Signaling	1: 1000
STAT2 rabbit	72604	Cell Signaling	1: 1000

Supplementary Table 2 Excitation wavelengths and corresponding filter sets used with Nikon Eclipse Ti2-Emicroscope.

Fluorophore	Excitation	Filter
eGFP/FITC	475 nM	Excitation: Chroma 89402x<br>Beamsplitter: Chroma 89402bs<br>Emission: Chroma 89402m
Atto594	575 nM	Excitation: Semrock 585/29 BrightLine HC<br>Beamsplitter: Chroma T610LPXR<br>Emission: Semrock 650/60 BrightLine HC
NucBlue (DAPI)	390 nM	Excitation: Chroma 89402x<br>Beamsplitter: Chroma 89402bs<br>Emission: Chroma 89402m

Gene and protein sequences for SNAP-ADAR versions used:

Gene & protein sequence of the SA1Q as expressed from engineered Flp-In-TREx 293 genome. E>Q site is highlighted in cyan.

```
                   10        20        30        40        50        60
1        ATGGACAAAGACTGCGAAATGAAGCGCACCACCCTGGATAGCCCTCTGGGCAAGCTGGAA
1         M  D  K  D  C  E  M  K  R  T  T  L  D  S  P  L  G  K  L  E

                   70        80        90       100       110       120
61       CTGTCTGGGTGCGAACAGGGCCTGCACCGTATCATCTTCCTGGGCAAAGGAACATCTGCC
21        L  S  G  C  E  Q  G  L  H  R  I  I  F  L  G  K  G  T  S  A

                  130       140       150       160       170       180
121      GCCGACGCCGTGGAAGTGCCTGCCCCAGCCGCCGTGCTGGGCGGACCAGAGCCACTGATG
41        A  D  A  V  E  V  P  A  P  A  A  V  L  G  G  P  E  P  L  M

                  190       200       210       220       230       240
181      CAGGCCACCGCCTGGCTCAACGCCTACTTTCACCAGCCTGAGGCCATCGAGGAGTTCCCT
61        Q  A  T  A  W  L  N  A  Y  F  H  Q  P  E  A  I  E  E  F  P

                  250       260       270       280       290       300
241      GTGCCAGCCCTGCACCACCCAGTGTTCCAGCAGGAGAGCTTTACCCGCCAGGTGCTGTGG
81        V  P  A  L  H  H  P  V  F  Q  Q  E  S  F  T  R  Q  V  L  W

                  310       320       330       340       350       360
301      AAACTGCTGAAAGTGGTGAAGTTCGGAGAGGTCATCAGCTACAGCCACCTGGCCGCCCTG
101       K  L  L  K  V  V  K  F  G  E  V  I  S  Y  S  H  L  A  A  L

                  370       380       390       400       410       420
361      GCCGGCAATCCCGCCGCCACCGCCGCCGTGAAAACCGCCCTGAGCGGAAATCCCGTGCCC
121       A  G  N  P  A  A  T  A  A  V  K  T  A  L  S  G  N  P  V  P

                  430       440       450       460       470       480
421      ATTCTGATCCCCTGCCACCGGGTGGTGCAGGGCGACCTGGACGTGGGGGGCTACGAGGGC
141       I  L  I  P  C  H  R  V  V  Q  G  D  L  D  V  G  G  Y  E  G

                  490       500       510       520       530       540
481      GGGCTCGCCGTGAAAGAGTGGCTGCTGGCCCACGAGGGCCACAGACTGGGCAAGCCTGGG
161       G  L  A  V  K  E  W  L  L  A  H  E  G  H  R  L  G  K  P  G

                  550       560       570       580       590       600
541      CTGGGTCCTGCAGGCGGAGGCGCGCCAGGGTCTGGCGGCGGCAGTAAGGCAGAACGCATG
181       L  G  P  A  G  G  G  A  P  G  S  G  G  G  S  K  A  E  R  M

                  610       620       630       640       650       660
601      GGTTTCACAGAGGTAACCCCAGTGACAGGGGCCAGTCTCAGAAGAACTATGCTCCTCCTC
201       G  F  T  E  V  T  P  V  T  G  A  S  L  R  R  T  M  L  L  L

                  670       680       690       700       710       720
661      TCAAGGTCCCCAGAAGCACAGCCAAAGACACTCCCTCTCACTGGCAGCACCTTCCATGAC
221       S  R  S  P  E  A  Q  P  K  T  L  P  L  T  G  S  T  F  H  D

                  730       740       750       760       770       780
721      CAGATAGCCATGCTGAGCCACCGGTGCTTCAACACTCTGACTAACAGCTTCCAGCCCTCC
241       Q  I  A  M  L  S  H  R  C  F  N  T  L  T  N  S  F  Q  P  S
```

```
             790       800       810       820       830       840
781    TTGCTCGGCCGCAAGATTCTGGCCGCCATCATTATGAAAAAAGACTCTGAGGACATGGGT
261     L  L  G  R  K  I  L  A  A  I  I  M  K  K  D  S  E  D  M  G

             850       860       870       880       890       900
841    GTCGTCGTCAGCTTGGGAACAGGGAATCGCTGTGTAAAAGGAGATTCTCTCAGCCTAAAA
281     V  V  V  S  L  G  T  G  N  R  C  V  K  G  D  S  L  S  L  K

             910       920       930       940       950       960
901    GGAGAAACTGTCAATGACTGCCATGCAGAAATAATCTCCCGGAGAGGCTTCATCAGGTTT
301     G  E  T  V  N  D  C  H  A  E  I  I  S  R  R  G  F  I  R  F

             970       980       990      1000      1010      1020
961    CTCTACAGTGAGTTAATGAAATACAACTCCCAGACTGCGAAGGATAGTATATTTGAACCT
321     L  Y  S  E  L  M  K  Y  N  S  Q  T  A  K  D  S  I  F  E  P

            1030      1040      1050      1060      1070      1080
1021   GCTAAGGGAGGAGAAAAGCTCCAAATAAAAAAGACTGTGTCATTCCATCTGTATATCAGC
341     A  K  G  G  E  K  L  Q  I  K  K  T  V  S  F  H  L  Y  I  S

            1090      1100      1110      1120      1130      1140
1081   ACTGCTCCGTGTGGAGATGGCGCCCTCTTTGACAAGTCCTGCAGCGACCGTGCTATGGAA
361     T  A  P  C  G  D  G  A  L  F  D  K  S  C  S  D  R  A  M  E

            1150      1160      1170      1180      1190      1200
1141   AGCACAGAATCCCGCCACTACCCTGTCTTCGAGAATCCCAAACAAGGAAAGCTCCGCACC
381     S  T  E  S  R  H  Y  P  V  F  E  N  P  K  Q  G  K  L  R  T

            1210      1220      1230      1240      1250      1260
1201   AAGGTGGAGAACGGACAAGGCACAATCCCTGTGGAATCCAGTGACATTGTGCCTACGTGG
401     K  V  E  N  G  Q  G  T  I  P  V  E  S  S  D  I  V  P  T  W

            1270      1280      1290      1300      1310      1320
1261   GATGGCATTCGGCTCGGGGAGAGACTCCGTACCATGTCCTGTAGTGACAAAATCCTACGC
421     D  G  I  R  L  G  E  R  L  R  T  M  S  C  S  D  K  I  L  R

            1330      1340      1350      1360      1370      1380
1321   TGGAACGTGCTGGGCCTGCAAGGGGCACTGTTGACCCACTTCCTGCAGCCCATTTATCTC
441     W  N  V  L  G  L  Q  G  A  L  L  T  H  F  L  Q  P  I  Y  L

            1390      1400      1410      1420      1430      1440
1381   AAATCTGTCACATTGGGTTACCTTTTCAGCCAAGGGCATCTGACCCGTGCTATTTGCTGT
461     K  S  V  T  L  G  Y  L  F  S  Q  G  H  L  T  R  A  I  C  C

            1450      1460      1470      1480      1490      1500
1441   CGTGTGACAAGAGATGGGAGTGCATTTGAGGATGGACTACGACATCCCTTTATTGTCAAC
481     R  V  T  R  D  G  S  A  F  E  D  G  L  R  H  P  F  I  V  N

            1510      1520      1530      1540      1550      1560
1501   CACCCCAAGGTTGGCAGAGTCAGCATATATGATTCCAAAAGGCAATCCGGGAAGACTAAG
501     H  P  K  V  G  R  V  S  I  Y  D  S  K  R  Q  S  G  K  T  K

            1570      1580      1590      1600      1610      1620
1561   GAGACAAGCGTCAACTGGTGTCTGGCTGATGGCTATGACCTGGAGATCCTGGACGGTACC
521     E  T  S  V  N  W  C  L  A  D  G  Y  D  L  E  I  L  D  G  T

            1630      1640      1650      1660      1670      1680
1621   AGAGGCACTGTGGATGGGCCACGGAATGAATTGTCCCGGGTCTCCAAAAAGAACATTTTT
541     R  G  T  V  D  G  P  R  N  E  L  S  R  V  S  K  K  N  I  F

            1690      1700      1710      1720      1730      1740
```

```
1681      CTTCTATTTAAGAAGCTCTGCTCCTTCCGTTACCGCAGGGATCTACTGAGACTCTCCTAT
561        L  L  F  K  K  L  C  S  F  R  Y  R  R  D  L  L  R  L  S  Y

                   1750      1760      1770      1780      1790      1800
1741      GGTGAGGCCAAGAAAGCTGCCCGTGACTACGAGACGGCCAAGAACTACTTCAAAAAAGGC
581        G  E  A  K  K  A  A  R  D  Y  E  T  A  K  N  Y  F  K  K  G

                   1810      1820      1830      1840      1850      1860
1801      CTGAAGGATATGGGCTATGGGAACTGGATTAGCAAACCCCAGGAGGAAAAGAACTTTTAT
601        L  K  D  M  G  Y  G  N  W  I  S  K  P  Q  E  E  K  N  F  Y

                   1870
1861      CTCTGCCCAGTA
621        L  C  P  V
```

Plasmid-maps & sequences:

Plasmid-map and sequence of PiggyBac plasmid SA1Q_XLone_Puro (pTS1086): The cyan highlighted site denotes E>Q mutation in hyperactive SNAP ADAR (SA1Q). The PiggyBac plasmid SA1_XLone_Puro (pTS1074) differs from this plasmid at this site with a GAA codon (Glu) instead of CAA codon(Gln).

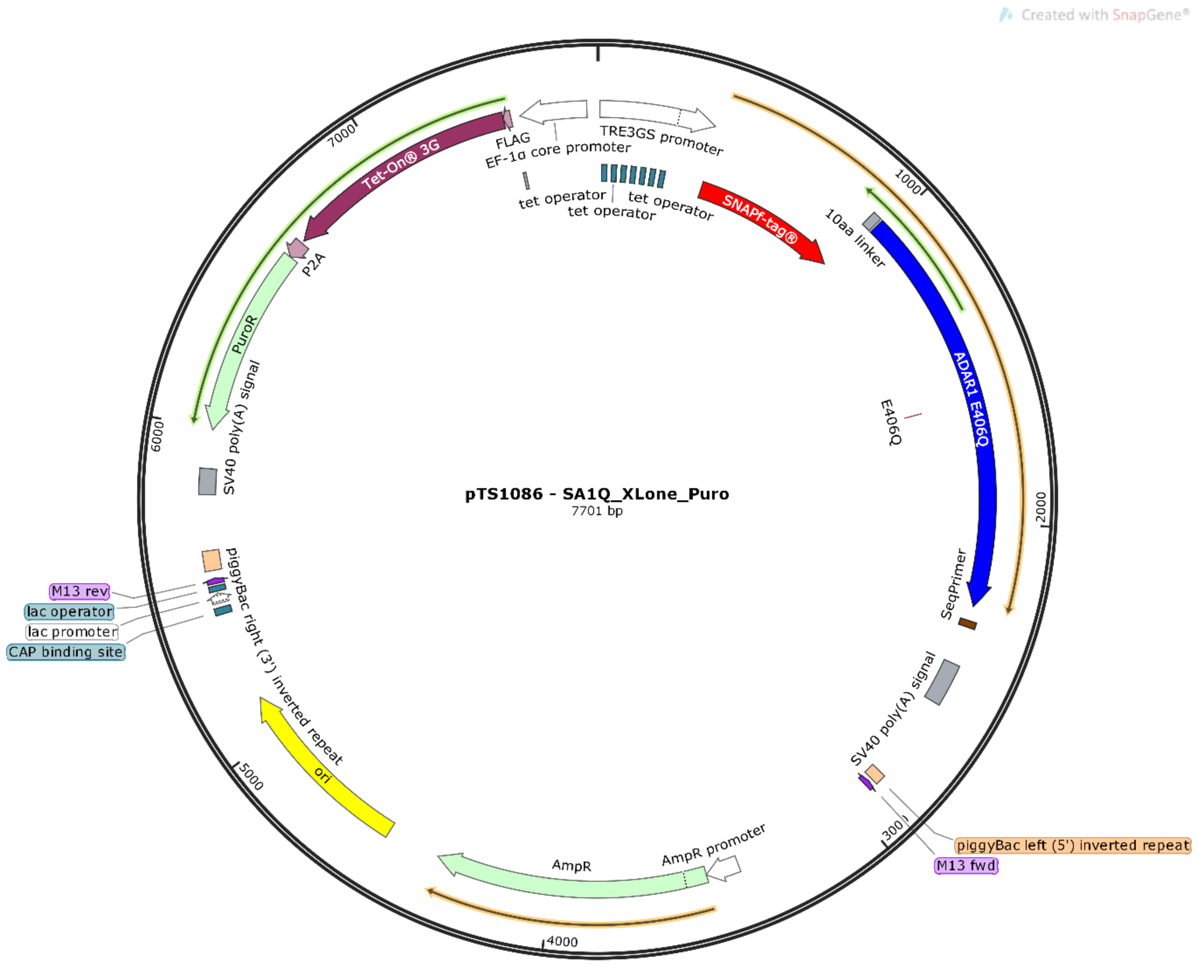

1	ATCACCTCGA	GTTTACTCCC	TATCAGTGAT	AGAGAACGTA	TGAAGAGTTT	ACTCCCTATC
61	AGTGATAGAG	AACGTATGCA	GACTTTACTC	CCTATCAGTG	ATAGAGAACG	TATAAGGAGT
121	TTACTCCCTA	TCAGTGATAG	AGAACGTATG	ACCAGTTTAC	TCCCTATCAG	TGATAGAGAA
181	CGTATCTACA	GTTTACTCCC	TATCAGTGAT	AGAGAACGTA	TATCCAGTTT	ACTCCCTATC
241	AGTGATAGAG	AACGTATAAG	CTTTGCTTAT	GTAAACCAGG	GCGCCTATAA	AAGAGTGCTG
301	ATTTTTTGAG	TAAACTTCAA	TTCCACAACA	CTTTTGTCTT	ATACCAACTT	TCCGTACCAC
361	TTCCTACCCT	CGTAAAGGTA	CCGCGGCCGC	CACCATGGAC	AAAGACTGCG	AAATGAAGCG
421	CACCACCCTG	GATAGCCCTC	TGGGCAAGCT	GGAACTGTCT	GGGTGCGAAC	AGGGCCTGCA
481	CCGTATCATC	TTCCTGGGCA	AAGGAACATC	TGCCGCCGAC	GCCGTGGAAG	TGCCTGCCCC
541	AGCCGCCGTG	CTGGGCGGAC	CAGAGCCACT	GATGCAGGCC	ACCGCCTGGC	TCAACGCCTA
601	CTTTCACCAG	CCTGAGGCCA	TCGAGGAGTT	CCCTGTGCCA	GCCCTGCACC	ACCCAGTGTT
661	CCAGCAGGAG	AGCTTTACCC	GCCAGGTGCT	GTGGAAACTG	CTGAAAGTGG	TGAAGTTCGG
721	AGAGGTCATC	AGCTACAGCC	ACCTGGCCGC	CCTGGCCGGC	AATCCCGCCG	CCACCGCCGC
781	CGTGAAAACC	GCCCTGAGCG	GAAATCCCGT	GCCCATTCTG	ATCCCCTGCC	ACCGGGTGGT
841	GCAGGGCGAC	CTGGACGTGG	GGGGCTACGA	GGGCGGGCTC	GCCGTGAAAG	AGTGGCTGCT
901	GGCCCACGAG	GGCCACAGAC	TGGGCAAGCC	TGGGCTGGGT	CCTGCAGGCG	GAGGCGCGCC

961 AGGGTCTGGC GGCGGCAGTA AGGCAGAACG CATGGGTTTC ACAGAGGTAA CCCCAGTGAC
1021 AGGGGCCAGT CTCAGAAGAA CTATGCTCCT CCTCTCAAGG TCCCCAGAAG CACAGCCAAA
1081 GACACTCCCT CTCACTGGCA GCACCTTCCA TGACCAGATA GCCATGCTGA GCCACCGGTG
1141 CTTCAACACT CTGACTAACA GCTTCCAGCC CTCCTTGCTC GGCCGCAAGA TTCTGGCCGC
1201 CATCATTATG AAAAAAGACT CTGAGGACAT GGGTGTCGTC GTCAGCTTGG GAACAGGGAA
1261 TCGCTGTGTA AAAGGAGATT CTCTCAGCCT AAAAGGAGAA ACTGTCAATG ACTGCCATGC
1321 AGAAATAATC TCCCGGAGAG GCTTCATCAG GTTTCTCTAC AGTGAGTTAA TGAAATACAA
1381 CTCCCAGACT GCGAAGGATA GTATATTTGA ACCTGCTAAG GGAGGAGAAA AGCTCCAAAT
1441 AAAAAAGACT GTGTCATTCC ATCTGTATAT CAGCACTGCT CCGTGTGGAG ATGGCGCCCT
1501 CTTTGACAAG TCCTGCAGCG ACCGTGCTAT GGAAAGCACA GAATCCCGCC ACTACCCTGT
1561 CTTCGAGAAT CCCAAACAAG GAAAGCTCCG CACCAAGGTG GAGAACGGAC AAGGCACAAT
1621 CCCTGTGGAA TCCAGTGACA TTGTGCCTAC GTGGGATGGC ATTCGGCTCG GGGAGAGACT
1681 CCGTACCATG TCCTGTAGTG ACAAAATCCT ACGCTGGAAC GTGCTGGGCC TGCAAGGGGC
1741 ACTGTTGACC CACTTCCTGC AGCCCATTTA TCTCAAATCT GTCACATTGG GTTACCTTTT
1801 CAGCCAAGGG CATCTGACCC GTGCTATTTG CTGTCGTGTG ACAAGAGATG GGAGTGCATT
1861 TGAGGATGGA CTACGACATC CCTTTATTGT CAACCACCCC AAGGTTGGCA GAGTCAGCAT
1921 ATATGATTCC AAAAGGCAAT CCGGGAAGAC TAAGGAGACA AGCGTCAACT GGTGTCTGGC
1981 TGATGGCTAT GACCTGGAGA TCCTGGACGG TACCAGAGGC ACTGTGGATG GGCCACGGAA
2041 TGAATTGTCC CGGGTCTCCA AAAAGAACAT TTTTCTTCTA TTTAAGAAGC TCTGCTCCTT
2101 CCGTTACCGC AGGGATCTAC TGAGACTCTC CTATGGTGAG GCCAAGAAAG CTGCCCGTGA
2161 CTACGAGACG GCCAAGAACT ACTTCAAAAA AGGCCTGAAG GATATGGGCT ATGGGAACTG
2221 GATTAGCAAA CCCCAGGAGG AAAAGAACTT TTATCTCTGC CCAGTATAAA TCGATTCTAG
2281 TAGACCACCT CCCCTGCGAG CTAAGCTGGA CAGCCAATGA CGGGTAAGAG AGTGACATTT
2341 TTCACTAACC TAAGACAGGA GGGCCGTCAG AGCTACTGCC TAATCCAAAG ACGGGTAAAA
2401 GTGATAAAAA TGTATCACTC CAACCTAAGA CAGGCGCAGC TTCCGAGGGA TTTGAGATCC
2461 AGACATGATA AGATACATTG ATGAGTTTGG ACAAACCAAA ACTAGAATGC AGTGAAAAAA
2521 ATGCCTTATT TGTGAAATTT GTGATGCTAT TGCCTTATTT GTAACCATTA TAAGCTGCAA
2581 TAAACAAGTT TGATATCTAT AACAAGAAAA TATATATATA ATAAGTTATC ACGTAAGTAG
2641 AACATGAAAT AACAATATAA TTATCGTATG AGTTAAATCT TAAAAGTCAC GTAAAAGATA
2701 ATCATGCGTC ATTTTGACTC ACGCGGTCGT TATAGTTCAA AATCAGTGAC ACTTACCGCA
2761 TTGACAAGCA CGCCTCACGG GAGCTCCAAG CGGCGACTGA GATGTCCTAA ATGCACAGCG
2821 ACGGATTCGC GCTATTTAGA AAGAGAGAGC AATATTTCAA GAATGCATGC GTCAATTTTA
2881 CGCAGACTAT CTTTCTAGGG TTAAGAATTC ACTGGCCGTC GTTTTACAAC GTCGTGACTG
2941 GGAAAACCCT GGCGTTACCC AACTTAATCG CCTTGCAGCA CATCCCCCTT TCGCCAGCTG
3001 GCGTAATAGC GAAGAGGCCC GCACCGATCG CCCTTCCCAA CAGTTGCGCA GCCTGAATGG
3061 CGAATGGCGC CTGATGCGGT ATTTTCTCCT TACGCATCTG TGCGGTATTT CACACCGCAT
3121 ATGGTGCACT CTCAGTACAA TCTGCTCTGA TGCCGCATAG TTAAGCCAGC CCCGACACCC
3181 GCCAACACCC GCTGACGCGC CCTGACGGGC TTGTCTGCTC CCGGCATCCG CTTACAGACA
3241 AGCTGTGACC GTCTCCGGGA GCTGCATGTG TCAGAGGTTT TCACCGTCAT CACCGAAACG
3301 CGCGAGACGA AAGGGCCTCG TGATACGCCT ATTTTTATAG GTTAATGTCA TGATAATAAT
3361 GGTTTCTTAG ACGTCAGGTG GCACTTTTCG GGGAAATGTG CGCGGAACCC CTATTTGTTT
3421 ATTTTTCTAA ATACATTCAA ATATGTATCC GCTCATGAGA CAATAACCCT GATAAATGCT
3481 TCAATAATAT TGAAAAAGGA AGAGTATGAG TATTCAACAT TTCCGTGTCG CCCTTATTCC
3541 CTTTTTTGCG GCATTTTGCC TTCCTGTTTT TGCTCACCCA GAAACGCTGG TGAAAGTAAA
3601 AGATGCTGAA GATCAGTTGG GTGCACGAGT GGGTTACATC GAACTGGATC TCAACAGCGG
3661 TAAGATCCTT GAGAGTTTTC GCCCCGAAGA ACGTTTTCCA ATGATGAGCA CTTTTAAAGT
3721 TCTGCTATGT GGCGCGGTAT TATCCCGTAT TGACGCCGGG CAAGAGCAAC TCGGTCGCCG
3781 CATACACTAT TCTCAGAATG ACTTGGTTGA GTACTCACCA GTCACAGAAA AGCATCTTAC
3841 GGATGGCATG ACAGTAAGAG AATTATGCAG TGCTGCCATA ACCATGAGTG ATAACACTGC
3901 GGCCAACTTA CTTCTGACAA CGATCGGAGG ACCGAAGGAG CTAACCGCTT TTTTGCACAA
3961 CATGGGGGAT CATGTAACTC GCCTTGATCG TTGGGAACCG GAGCTGAATG AAGCCATACC
4021 AAACGACGAG CGTGACACCA CGATGCCTGT AGCAATGGCA ACAACGTTGC GCAAACTATT
4081 AACTGGCGAA CTACTTACTC TAGCTTCCCG GCAACAATTA ATAGACTGGA TGGAGGCGGA
4141 TAAAGTTGCA GGACCACTTC TGCGCTCGGC CCTTCCGGCT GGCTGGTTTA TTGCTGATAA
4201 ATCTGGAGCC GGTGAGCGTG GGTCTCGCGG TATCATTGCA GCACTGGGGC CAGATGGTAA
4261 GCCCTCCCGT ATCGTAGTTA TCTACACGAC GGGGAGTCAG GCAACTATGG ATGAACGAAA
4321 TAGACAGATC GCTGAGATAG GTGCCTCACT GATTAAGCAT TGGTAACTGT CAGACCAAGT
4381 TTACTCATAT ATACTTTAGA TTGATTTAAA ACTTCATTTT TAATTTAAAA GGATCTAGGT
4441 GAAGATCCTT TTTGATAATC TCATGACCAA AATCCCTTAA CGTGAGTTTT CGTTCCACTG
4501 AGCGTCAGAC CCCGTAGAAA AGATCAAAGG ATCTTCTTGA GATCCTTTTT TTCTGCGCGT
4561 AATCTGCTGC TTGCAAACAA AAAAACCACC GCTACCAGCG GTGGTTTGTT TGCCGGATCA

4621 AGAGCTACCA ACTCTTTTTC CGAAGGTAAC TGGCTTCAGC AGAGCGCAGA TACCAAATAC
4681 TGTTCTTCTA GTGTAGCCGT AGTTAGGCCA CCACTTCAAG AACTCTGTAG CACCGCCTAC
4741 ATACCTCGCT CTGCTAATCC TGTTACCAGT GGCTGCTGCC AGTGGCGATA AGTCGTGTCT
4801 TACCGGGTTG GACTCAAGAC GATAGTTACC GGATAAGGCG CAGCGGTCGG GCTGAACGGG
4861 GGGTTCGTGC ACACAGCCCA GCTTGGAGCG AACGACCTAC ACCGAACTGA GATACCTACA
4921 GCGTGAGCTA TGAGAAAGCG CCACGCTTCC CGAAGGGAGA AAGGCGGACA GGTATCCGGT
4981 AAGCGGCAGG GTCGGAACAG GAGAGCGCAC GAGGGAGCTT CCAGGGGGAA ACGCCTGGTA
5041 TCTTTATAGT CCTGTCGGGT TTCGCCACCT CTGACTTGAG CGTCGATTTT TGTGATGCTC
5101 GTCAGGGGGG CGGAGCCTAT GGAAAAACGC CAGCAACGCG GCCTTTTTAC GGTTCCTGGC
5161 CTTTTGCTGG CCTTTTGCTC ACATGTTCTT TCCTGCGTTA TCCCCTGATT CTGTGGATAA
5221 CCGTATTACC GCCTTTGAGT GAGCTGATAC CGCTCGCCGC AGCCGAACGA CCGAGCGCAG
5281 CGAGTCAGTG AGCGAGGAAG CGGAAGAGCG CCCAATACGC AAACCGCCTC TCCCCGCGCG
5341 TTGGCCGATT CATTAATGCA GCTGGCACGA CAGGTTTCCC GACTGGAAAG CGGGCAGTGA
5401 GCGCAACGCA ATTAATGTGA GTTAGCTCAC TCATTAGGCA CCCCAGGCTT TACACTTTAT
5461 GCTTCCGGCT CGTATGTTGT GTGGAATTGT GAGCGGATAA CAATTTCACA CAGGAAACAG
5521 CTATGACCAT GATTACGCCA AGGTCGACTT AACCCTAGAA AGATAATCAT ATTGTGACGT
5581 ACGTTAAAGA TAATCATGCG TAAAATTGAC GCATGTGTTT TATCGGTCTG TATATCGAGG
5641 TTTATTTATT AATTTGAATA GATATTAAGT TTTATTATAT TTACACTTAC ATACTAATAA
5701 TAAATTCAAC AAACAATTTA TTTATGTTTA TTTATTTATT AAAAAAAAAC AAAAACTCAA
5761 AATTTCTTCT ATAAAGTAAC AAAACTTTTA GCAGTGAAAA AAATGCTTTA TTTGTGAAAT
5821 TTGTGATGCT ATTGCTTTAT TTGTAACCAT TATAAGCTGC AATAAACAAG TTAACAACAA
5881 CAATTGCATT CATTTTATGT TTCAGGTTCA GGGGGAGGTG TGGGAGGTTT TTTAAAGCAA
5941 GTAAAACCTC TACAAATGTG GTATGGCTGA TTATGATCCT CTGGAGATCC TAGGCTAGGC
6001 ACCGGGCTTG CGGGTCATGC ACCAGGTGCG CGGTCCTTCG GGCACCTCGA CGTCGGCGGT
6061 GACGGTGAAG CCGAGCCGCT CGTAGAAGGG GAGGTTGCGG GGCGCGGAGG TCTCCAGGAA
6121 GGCGGGCACC CCGGCGCGCT CGGCCGCCTC CACTCCGGGG AGCACGACGG CGCTGCCCAG
6181 ACCCTTGCCC TGGTGGTCGG GCGAGACGCC GACGGTGGCC AGGAACCACG CGGGCTCCTT
6241 GGGCCGGTGC GGCGCCAGGA GGCCTTCCAT CTGTTGCTGC GCGGCCAGCC GGGAACCGCT
6301 CAACTCGGCC ATGCGCGGGC CGATCTCGGC GAACACCGCC CCCGCTTCGA CGCTCTCCGG
6361 CGTGGTCCAG ACCGCCACCG CGGCGCCGTC GTCCGCGACC CACACCTTGC CGATGTCGAG
6421 CCCGACGCGC GTGAGGAAGA GTTCTTGCAG CTCGGTGACC CGCTCGATGT GGCGGTCCGG
6481 ATCGACGGTG TGGCGCGTGG CGGGGTAGTC GGCGAACGCG GCGGCGAGGG TGCGTACGGC
6541 CCTGGGGACG TCGTCGCGGG TGGCGAGGCG CACCGTGGGC TTGTACTCGG TCATGGGGCC
6601 GGGGTTCTCC TCCACGTCGC CGGCCTGCTT CAGCAGGCTG AAGTTGGTGG CGCCGCTGCC
6661 CCCGGGGAGC ATGTCAAGGT CAAAATCGTC AAGAGCGTCA GCAGGCAGCA TATCAAGGTC
6721 AAAGTCGTCA AGGGCATCGG CTGGGAGCAT GTCTAAGTCA AAATCGTCAA GGGCGTCGGT
6781 CGGCCCGCCG CTTTCGCACT TTAGCTGTTT CTCCAGGCCA CATATGATTA GTTCCAGGCC
6841 GAAAAGGAAG GCAGGTTCGG CTCCCTGCCG GTCGAACAGC TCAATTGCTT GTTTCAGAAG
6901 TGGGGGCATA GAATCGGTGG TAGGTGTCTC TCTTTCCTCT TTTGCTACTT GATGCTCCTG
6961 TTCCTCCAAT ACGCAGCCCA GTGTAAAGTG GCCCACGGCG GACAGAGCGT ACAGTGCGTT
7021 CTCCAGGGAG AAGCCTTGCT GACACAGGAA CGCGAGCTGA TTTTCCAGGG TTTCGTACTG
7081 TTTCTCTGTT GGGCGGGTGC CGAGATGCAC TTTAGCCCCG TCGCGATGTG AGAGGAGAGC
7141 ACAGCGGTAT GACTTGGCGT TGTTCCGCAG AAAGTCTTGC CATGACTCGC CTTCCAGGGG
7201 GCAGGAGTGG GTATGATGCC TGTCCAGCAT CTCGATTGGC AGGGCATCGA GCAGGGCCCG
7261 CTTGTTCTTC ACGTGCCAGT ACAGGGTAGG CTGCTCAACT CCCAGCTTTT GAGCGAGTTT
7321 CCTTGTCGTC AGGCCTTCGA TACCGACTCC ATTGAGTAAT TCCAGAGCAG AGTTTATGAC
7381 TTTGCTCTTG TCCAGTCTAG ACATCTTATC GTCATCGTCT TTGTAATCCA TGGTGGCGGA
7441 TCCCGCGTCA CGACACCTGT GTTCTGGCGG CAAACCCGTT GCGAAAAAGA ACGTTCACGG
7501 CGACTACTGC ACTTATATAC GGTTCTCCCC CACCCTCGGG AAAAAGGCGG AGCCAGTACA
7561 CGACATCACT TTCCCAGTTT ACCCCGCGCC ACCTTCTCTA GGCACCGGTT CAATTGCCGA
7621 CCCCTCCCCC CAACTTCTCG GGGACTGTGG GCGATGTGCG CTCTGCCCAC TGACGGGCAC
7681 CGGAGCCACT CGAGTGGAAT T

Plasmid-map and sequence of PiggyBac plasmid SA1Q_XLone_BSD (pTS1018): The cyan highlighted site denotes E>Q mutation in hyperactive SNAP ADAR (SA1Q). The PiggyBac plasmid SA1_XLone_BSD (pTS990) differs from this plasmid at this site with a GAA codon (Glu) instead of CAA codon (Gln).

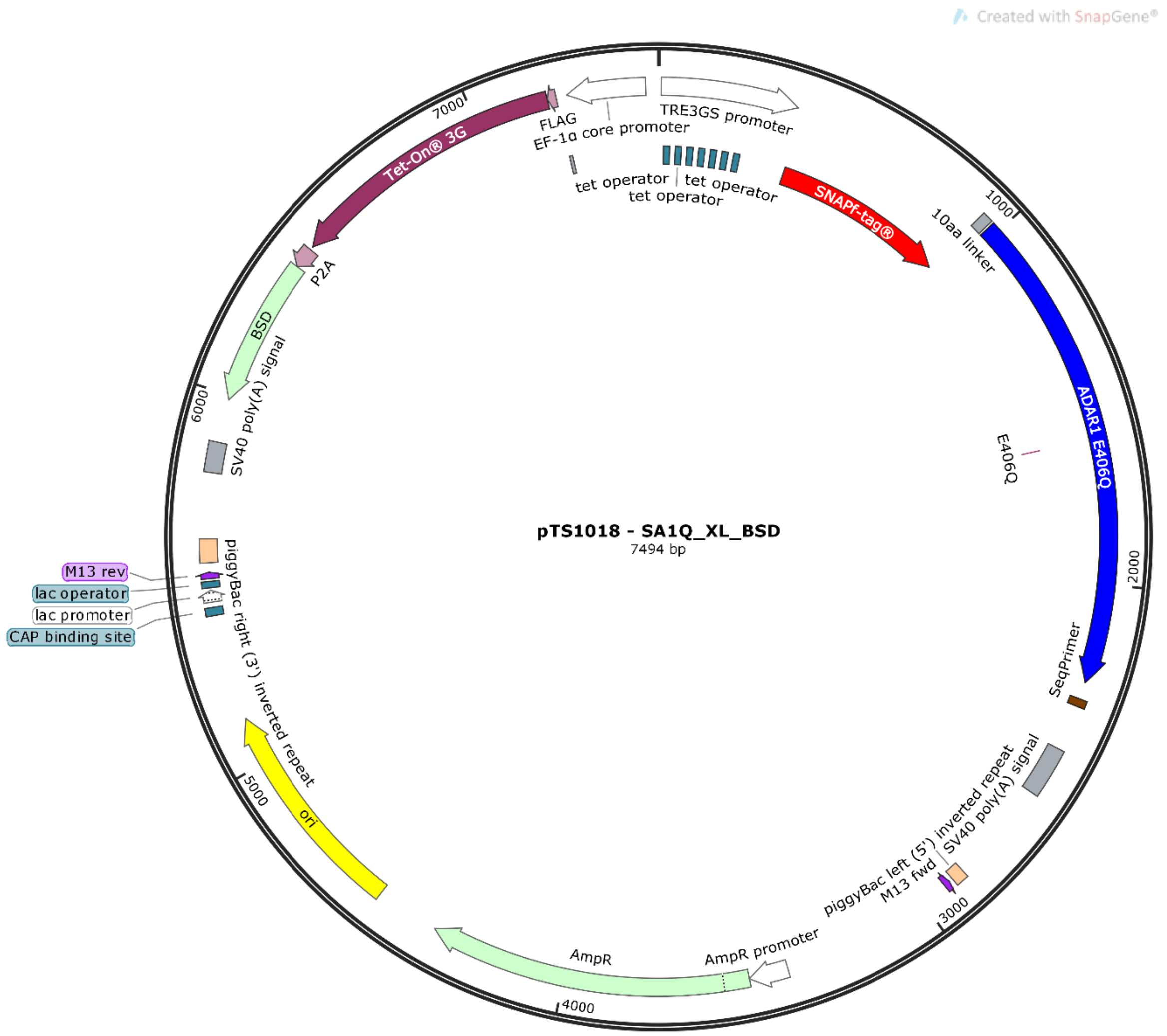


```
1      ATCACCTCGA GTTTACTCCC TATCAGTGAT AGAGAACGTA TGAAGAGTTT ACTCCCTATC
61     AGTGATAGAG AACGTATGCA GACTTTACTC CCTATCAGTG ATAGAGAACG TATAAGGAGT
121    TTACTCCCTA TCAGTGATAG AGAACGTATG ACCAGTTTAC TCCCTATCAG TGATAGAGAA
181    CGTATCTACA GTTTACTCCC TATCAGTGAT AGAGAACGTA TATCCAGTTT ACTCCCTATC
241    AGTGATAGAG AACGTATAAG CTTTGCTTAT GTAAACCAGG GCGCCTATAA AAGAGTGCTG
301    ATTTTTTGAG TAAACTTCAA TTCCACAACA CTTTTGTCTT ATACCAACTT TCCGTACCAC
361    TTCCTACCCT CGTAAAGGTA CCGCGGCCGC CACCATGGAC AAAGACTGCG AAATGAAGCG
421    CACCACCCTG GATAGCCCTC TGGGCAAGCT GGAACTGTCT GGGTGCGAAC AGGGCCTGCA
481    CCGTATCATC TTCCTGGGCA AAGGAACATC TGCCGCCGAC GCCGTGGAAG TGCCTGCCCC
541    AGCCGCCGTG CTGGGCGGAC CAGAGCCACT GATGCAGGCC ACCGCCTGGC TCAACGCCTA
601    CTTTCACCAG CCTGAGGCCA TCGAGGAGTT CCCTGTGCCA GCCCTGCACC ACCCAGTGTT
661    CCAGCAGGAG AGCTTTACCC GCCAGGTGCT GTGGAAACTG CTGAAAGTGG TGAAGTTCGG
721    AGAGGTCATC AGCTACAGCC ACCTGGCCGC CCTGGCCGGC AATCCCGCCG CCACCGCCGC
781    CGTGAAAACC GCCCTGAGCG GAAATCCCGT GCCCATTCTG ATCCCCTGCC ACCGGGTGGT
841    GCAGGGCGAC CTGGACGTGG GGGGCTACGA GGGCGGGCTC GCCGTGAAAG AGTGGCTGCT
901    GGCCCACGAG GGCCACAGAC TGGGCAAGCC TGGGCTGGGT CCTGCAGGCG GAGGCGCGCC
```

961	AGGGTCTGGC	GGCGGCAGTA	AGGCAGAACG	CATGGGTTTC	ACAGAGGTAA	CCCCAGTGAC
1021	AGGGGCCAGT	CTCAGAAGAA	CTATGCTCCT	CCTCTCAAGG	TCCCCAGAAG	CACAGCCAAA
1081	GACACTCCCT	CTCACTGGCA	GCACCTTCCA	TGACCAGATA	GCCATGCTGA	GCCACCGGTG
1141	CTTCAACACT	CTGACTAACA	GCTTCCAGCC	CTCCTTGCTC	GGCCGCAAGA	TTCTGGCCGC
1201	CATCATTATG	AAAAAAGACT	CTGAGGACAT	GGGTGTCGTC	GTCAGCTTGG	GAACAGGGAA
1261	TCGCTGTGTA	AAAGGAGATT	CTCTCAGCCT	AAAAGGAGAA	ACTGTCAATG	ACTGCCATGC
1321	AGAAATAATC	TCCCGGAGAG	GCTTCATCAG	GTTTCTCTAC	AGTGAGTTAA	TGAAATACAA
1381	CTCCCAGACT	GCGAAGGATA	GTATATTTGA	ACCTGCTAAG	GGAGGAGAAA	AGCTCCAAAT
1441	AAAAAAGACT	GTGTCATTCC	ATCTGTATAT	CAGCACTGCT	CCGTGTGGAG	ATGGCGCCCT
1501	CTTTGACAAG	TCCTGCAGCG	ACCGTGCTAT	GGAAAGCACA	GAATCCCGCC	ACTACCCTGT
1561	CTTCGAGAAT	CCCAAACAAG	GAAAGCTCCG	CACCAAGGTG	GAGAACGGAC	AAGGCACAAT
1621	CCCTGTGGAA	TCCAGTGACA	TTGTGCCTAC	GTGGGATGGC	ATTCGGCTCG	GGGAGAGACT
1681	CCGTACCATG	TCCTGTAGTG	ACAAAATCCT	ACGCTGGAAC	GTGCTGGGCC	TGCAAGGGGC
1741	ACTGTTGACC	CACTTCCTGC	AGCCCATTTA	TCTCAAATCT	GTCACATTGG	GTTACCTTTT
1801	CAGCCAAGGG	CATCTGACCC	GTGCTATTTG	CTGTCGTGTG	ACAAGAGATG	GGAGTGCATT
1861	TGAGGATGGA	CTACGACATC	CCTTTATTGT	CAACCACCCC	AAGGTTGGCA	GAGTCAGCAT
1921	ATATGATTCC	AAAAGGCAAT	CCGGGAAGAC	TAAGGAGACA	AGCGTCAACT	GGTGTCTGGC
1981	TGATGGCTAT	GACCTGGAGA	TCCTGGACGG	TACCAGAGGC	ACTGTGGATG	GGCCACGGAA
2041	TGAATTGTCC	CGGGTCTCCA	AAAAGAACAT	TTTTCTTCTA	TTTAAGAAGC	TCTGCTCCTT
2101	CCGTTACCGC	AGGGATCTAC	TGAGACTCTC	CTATGGTGAG	GCCAAGAAAG	CTGCCCGTGA
2161	CTACGAGACG	GCCAAGAACT	ACTTCAAAAA	AGGCCTGAAG	GATATGGGCT	ATGGGAACTG
2221	GATTAGCAAA	CCCCAGGAGG	AAAAGAACTT	TTATCTCTGC	CCAGTATAAA	TCGATTCTAG
2281	TAGACCACCT	CCCCTGCGAG	CTAAGCTGGA	CAGCCAATGA	CGGGTAAGAG	AGTGACATTT
2341	TTCACTAACC	TAAGACAGGA	GGGCCGTCAG	AGCTACTGCC	TAATCCAAAG	ACGGGTAAAA
2401	GTGATAAAAA	TGTATCACTC	CAACCTAAGA	CAGGCGCAGC	TTCCGAGGGA	TTTGAGATCC
2461	AGACATGATA	AGATACATTG	ATGAGTTTGG	ACAAACCAAA	ACTAGAATGC	AGTGAAAAAA
2521	ATGCCTTATT	TGTGAAATTT	GTGATGCTAT	TGCCTTATTT	GTAACCATTA	TAAGCTGCAA
2581	TAAACAAGTT	TGATATCTAT	AACAAGAAAA	TATATATATA	ATAAGTTATC	ACGTAAGTAG
2641	AACATGAAAT	AACAATATAA	TTATCGTATG	AGTTAAATCT	TAAAAGTCAC	GTAAAAGATA
2701	ATCATGCGTC	ATTTTGACTC	ACGCGGTCGT	TATAGTTCAA	AATCAGTGAC	ACTTACCGCA
2761	TTGACAAGCA	CGCCTCACGG	GAGCTCCAAG	CGGCGACTGA	GATGTCCTAA	ATGCACAGCG
2821	ACGGATTCGC	GCTATTTAGA	AAGAGAGAGC	AATATTTCAA	GAATGCATGC	GTCAATTTTA
2881	CGCAGACTAT	CTTTCTAGGG	TTAAGAATTC	ACTGGCCGTC	GTTTTACAAC	GTCGTGACTG
2941	GGAAAACCCT	GGCGTTACCC	AACTTAATCG	CCTTGCAGCA	CATCCCCCTT	TCGCCAGCTG
3001	GCGTAATAGC	GAAGAGGCCC	GCACCGATCG	CCCTTCCCAA	CAGTTGCGCA	GCCTGAATGG
3061	CGAATGGCGC	CTGATGCGGT	ATTTTCTCCT	TACGCATCTG	TGCGGTATTT	CACACCGCAT
3121	ATGGTGCACT	CTCAGTACAA	TCTGCTCTGA	TGCCGCATAG	TTAAGCCAGC	CCCGACACCC
3181	GCCAACACCC	GCTGACGCGC	CCTGACGGGC	TTGTCTGCTC	CCGGCATCCG	CTTACAGACA
3241	AGCTGTGACC	GTCTCCGGGA	GCTGCATGTG	TCAGAGGTTT	TCACCGTCAT	CACCGAAACG
3301	CGCGAGACGA	AAGGGCCTCG	TGATACGCCT	ATTTTTATAG	GTTAATGTCA	TGATAATAAT
3361	GGTTTCTTAG	ACGTCAGGTG	GCACTTTTCG	GGGAAATGTG	CGCGGAACCC	CTATTTGTTT
3421	ATTTTTCTAA	ATACATTCAA	ATATGTATCC	GCTCATGAGA	CAATAACCCT	GATAAATGCT
3481	TCAATAATAT	TGAAAAAGGA	AGAGTATGAG	TATTCAACAT	TTCCGTGTCG	CCCTTATTCC
3541	CTTTTTTGCG	GCATTTTGCC	TTCCTGTTTT	TGCTCACCCA	GAAACGCTGG	TGAAAGTAAA
3601	AGATGCTGAA	GATCAGTTGG	GTGCACGAGT	GGGTTACATC	GAACTGGATC	TCAACAGCGG
3661	TAAGATCCTT	GAGAGTTTTC	GCCCCGAAGA	ACGTTTTCCA	ATGATGAGCA	CTTTTAAAGT
3721	TCTGCTATGT	GGCGCGGTAT	TATCCCGTAT	TGACGCCGGG	CAAGAGCAAC	TCGGTCGCCG
3781	CATACACTAT	TCTCAGAATG	ACTTGGTTGA	GTACTCACCA	GTCACAGAAA	AGCATCTTAC
3841	GGATGGCATG	ACAGTAAGAG	AATTATGCAG	TGCTGCCATA	ACCATGAGTG	ATAACACTGC
3901	GGCCAACTTA	CTTCTGACAA	CGATCGGAGG	ACCGAAGGAG	CTAACCGCTT	TTTTGCACAA
3961	CATGGGGGAT	CATGTAACTC	GCCTTGATCG	TTGGGAACCG	GAGCTGAATG	AAGCCATACC
4021	AAACGACGAG	CGTGACACCA	CGATGCCTGT	AGCAATGGCA	ACAACGTTGC	GCAAACTATT
4081	AACTGGCGAA	CTACTTACTC	TAGCTTCCCG	GCAACAATTA	ATAGACTGGA	TGGAGGCGGA
4141	TAAAGTTGCA	GGACCACTTC	TGCGCTCGGC	CCTTCCGGCT	GGCTGGTTTA	TTGCTGATAA
4201	ATCTGGAGCC	GGTGAGCGTG	GGTCTCGCGG	TATCATTGCA	GCACTGGGGC	CAGATGGTAA
4261	GCCCTCCCGT	ATCGTAGTTA	TCTACACGAC	GGGGAGTCAG	GCAACTATGG	ATGAACGAAA
4321	TAGACAGATC	GCTGAGATAG	GTGCCTCACT	GATTAAGCAT	TGGTAACTGT	CAGACCAAGT
4381	TTACTCATAT	ATACTTTAGA	TTGATTTAAA	ACTTCATTTT	TAATTTAAAA	GGATCTAGGT
4441	GAAGATCCTT	TTTGATAATC	TCATGACCAA	AATCCCTTAA	CGTGAGTTTT	CGTTCCACTG
4501	AGCGTCAGAC	CCCGTAGAAA	AGATCAAAGG	ATCTTCTTGA	GATCCTTTTT	TTCTGCGCGT
4561	AATCTGCTGC	TTGCAAACAA	AAAAACCACC	GCTACCAGCG	GTGGTTTGTT	TGCCGGATCA

4621 AGAGCTACCA ACTCTTTTTC CGAAGGTAAC TGGCTTCAGC AGAGCGCAGA TACCAAATAC
4681 TGTTCTTCTA GTGTAGCCGT AGTTAGGCCA CCACTTCAAG AACTCTGTAG CACCGCCTAC
4741 ATACCTCGCT CTGCTAATCC TGTTACCAGT GGCTGCTGCC AGTGGCGATA AGTCGTGTCT
4801 TACCGGGTTG GACTCAAGAC GATAGTTACC GGATAAGGCG CAGCGGTCGG GCTGAACGGG
4861 GGGTTCGTGC ACACAGCCCA GCTTGGAGCG AACGACCTAC ACCGAACTGA GATACCTACA
4921 GCGTGAGCTA TGAGAAAGCG CCACGCTTCC CGAAGGGAGA AAGGCGGACA GGTATCCGGT
4981 AAGCGGCAGG GTCGGAACAG GAGAGCGCAC GAGGGAGCTT CCAGGGGGAA ACGCCTGGTA
5041 TCTTTATAGT CCTGTCGGGT TTCGCCACCT CTGACTTGAG CGTCGATTTT TGTGATGCTC
5101 GTCAGGGGGG CGGAGCCTAT GGAAAAACGC CAGCAACGCG GCCTTTTTAC GGTTCCTGGC
5161 CTTTTGCTGG CCTTTTGCTC ACATGTTCTT TCCTGCGTTA TCCCCTGATT CTGTGGATAA
5221 CCGTATTACC GCCTTTGAGT GAGCTGATAC CGCTCGCCGC AGCCGAACGA CCGAGCGCAG
5281 CGAGTCAGTG AGCGAGGAAG CGGAAGAGCG CCCAATACGC AAACCGCCTC TCCCCGCGCG
5341 TTGGCCGATT CATTAATGCA GCTGGCACGA CAGGTTTCCC GACTGGAAAG CGGGCAGTGA
5401 GCGCAACGCA ATTAATGTGA GTTAGCTCAC TCATTAGGCA CCCCAGGCTT TACACTTTAT
5461 GCTTCCGGCT CGTATGTTGT GTGGAATTGT GAGCGGATAA CAATTTCACA CAGGAAACAG
5521 CTATGACCAT GATTACGCCA AGGTCGACTT AACCCTAGAA AGATAATCAT ATTGTGACGT
5581 ACGTTAAAGA TAATCATGCG TAAAATTGAC GCATGTGTTT TATCGGTCTG TATATCGAGG
5641 TTTATTTATT AATTTGAATA GATATTAAGT TTTATTATAT TTACACTTAC ATACTAATAA
5701 TAAATTCAAC AAACAATTTA TTTATGTTTA TTTATTTATT AAAAAAAAAC AAAAACTCAA
5761 AATTTCTTCT ATAAAGTAAC AAAACTTTTA GCAGTGAAAA AAATGCTTTA TTTGTGAAAT
5821 TTGTGATGCT ATTGCTTTAT TTGTAACCAT TATAAGCTGC AATAAACAAG TTAACAACAA
5881 CAATTGCATT CATTTTATGT TTCAGGTTCA GGGGGAGGTG TGGGAGGTTT TTTAAAGCAA
5941 GTAAAACCTC TACAAATGTG GTATGGCTGA TTATGATCCT CTGGAGATTT AGCCCTCCCA
6001 CACATAACCA GAGGGCAGCA ATTCACGAAT CCCAACTGCC GTCGGCTGTC CATCACTGTC
6061 CTTCACTATG GCTTTGATCC CAGGATGCAG ATCGAGAAGC ACCTGTCGGC ACCGTCCGCA
6121 GGGGCTCAAG ATGCCCCTGT TCTCATTTCC GATCGCGACG ATACAAGTCA GGTTGCCAGC
6181 TGCCGCAGCA GCAGCAGTGC CCAGCACCAC GAGTTCTGCA CAAGGTCCCC CAGTAAAATG
6241 ATATACATTG ACACCAGTGA AGATGCGGCC GTCGCTAGAG AGAGCTGCGC TGGCGACGCT
6301 GTAGTCTTCA GAGATGGGGA TGCTGTTGAT TGTAGCCGTT GCTCTTTCAA TGAGGGTGGA
6361 TTCTTCTTGA GACAAAGGCT TGGCCATGGG GCCGGGGTTC TCCTCCACGT CGCCGGCCTG
6421 CTTCAGCAGG CTGAAGTTGG TGGCGCCGCT GCCCCCGGGG AGCATGTCAA GGTCAAAATC
6481 GTCAAGAGCG TCAGCAGGCA GCATATCAAG GTCAAAGTCG TCAAGGGCAT CGGCTGGGAG
6541 CATGTCTAAG TCAAAATCGT CAAGGGCGTC GGTCGGCCCG CCGCTTTCGC ACTTTAGCTG
6601 TTTCTCCAGG CCACATATGA TTAGTTCCAG GCCGAAAAGG AAGGCAGGTT CGGCTCCCTG
6661 CCGGTCGAAC AGCTCAATTG CTTGTTTCAG AAGTGGGGGC ATAGAATCGG TGGTAGGTGT
6721 CTCTCTTTCC TCTTTTGCTA CTTGATGCTC CTGTTCCTCC AATACGCAGC CCAGTGTAAA
6781 GTGGCCCACG GCGGACAGAG CGTACAGTGC GTTCTCCAGG GAGAAGCCTT GCTGACACAG
6841 GAACGCGAGC TGATTTTCCA GGGTTTCGTA CTGTTTCTCT GTTGGGCGGG TGCCGAGATG
6901 CACTTTAGCC CCGTCGCGAT GTGAGAGGAG AGCACAGCGG TATGACTTGG CGTTGTTCCG
6961 CAGAAAGTCT TGCCATGACT CGCCTTCCAG GGGGCAGGAG TGGGTATGAT GCCTGTCCAG
7021 CATCTCGATT GGCAGGGCAT CGAGCAGGGC CCGCTTGTTC TTCACGTGCC AGTACAGGGT
7081 AGGCTGCTCA ACTCCCAGCT TTTGAGCGAG TTTCCTTGTC GTCAGGCCTT CGATACCGAC
7141 TCCATTGAGT AATTCCAGAG CAGAGTTTAT GACTTTGCTC TTGTCCAGTC TAGACATCTT
7201 ATCGTCATCG TCTTTGTAAT CCATGGTGGC GGATCCCGCG TCACGACACC TGTGTTCTGG
7261 CGGCAAACCC GTTGCGAAAA AGAACGTTCA CGGCGACTAC TGCACTTATA TACGGTTCTC
7321 CCCCACCCTC GGGAAAAAGG CGGAGCCAGT ACACGACATC ACTTTCCCAG TTTACCCCGC
7381 GCCACCTTCT CTAGGCACCG GTTCAATTGC CGACCCCTCC CCCCAACTTC TCGGGGACTG
7441 TGGGCGATGT GCGCTCTGCC CACTGACGGG CACCGGAGCC ACTCGAGTGG AATT

Plasmid-map and sequence of SNAP AdV SA1Q, minimal CMV (pTS2396):

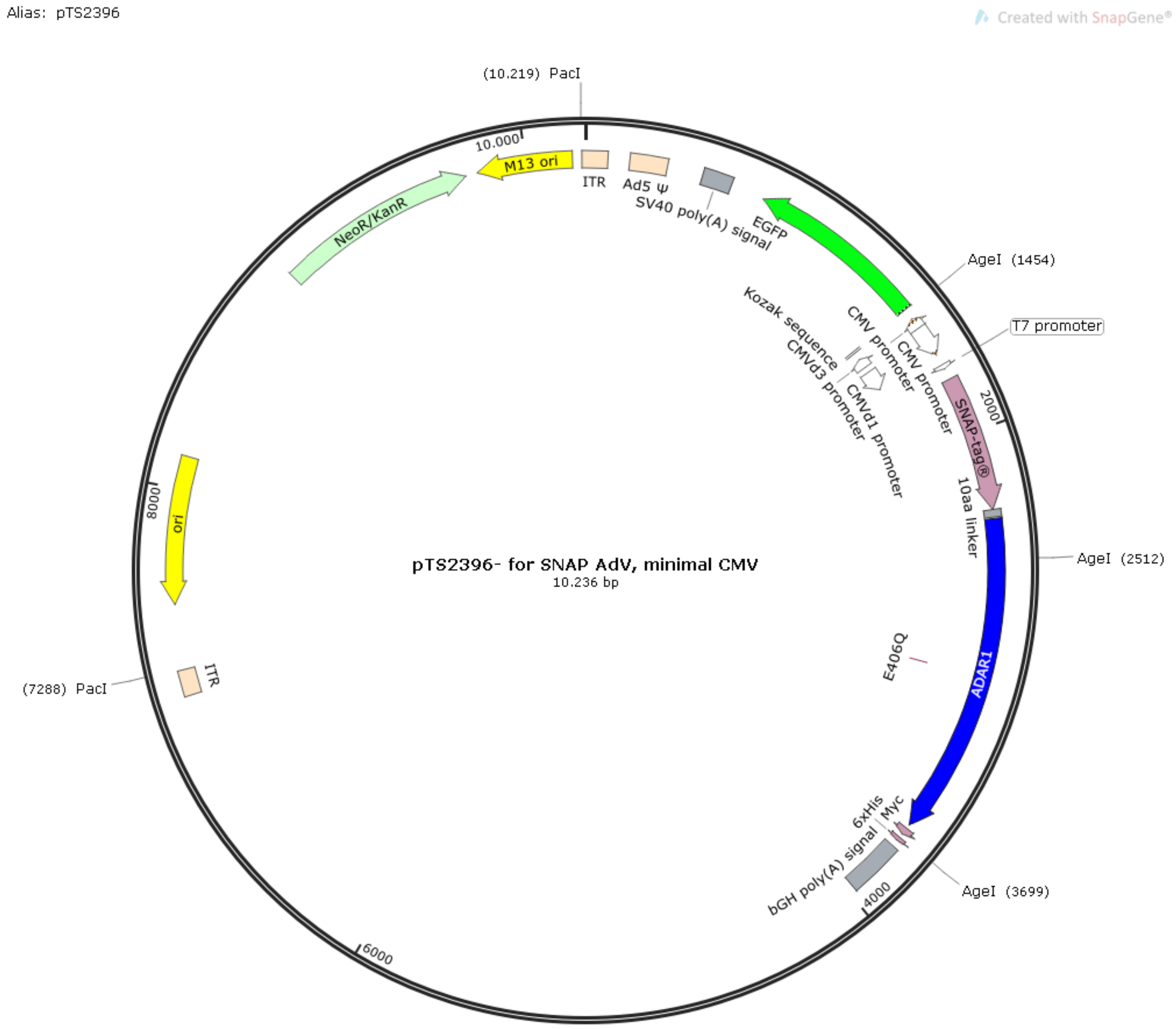


```
1       TACCTTATTT TGGATTGAAG CCAATATGAT AATGAGGGGG TGGAGTTTGT GACGTGGCGC
61      GGGGCGTGGG AACGGGGCGG GTGACGTAGT AGTGTGGCGG AAGTGTGATG TTGCAAGTGT
121     GGCGGAACAC ATGTAAGCGA CGGATGTGGC AAAAGTGACG TTTTTGGTGT GCGCCGGTGT
181     ACACAGGAAG TGACAATTTT CGCGCGGTTT TAGGCGGATG TTGTAGTAAA TTTGGGCGTA
241     ACCGAGTAAG ATTTGGCCAT TTTCGCGGGA AAACTGAATA AGAGGAAGTG AAATCTGAAT
301     AATTTTGTGT TACTCATAGC GCGTAATATT TGTCTAGGGC CGCGGGGACT TTGACCGTTT
361     ACGTGGAGAC TCGCCCAGGT GTTTTTCTCA GGTGTTTTCC GCGTTCCGGG TCAAAGTTGG
421     CGTTTTATTA TTATAGTCAG TCGAGTCTAG CAAGCTAGCT TGATCGCGTT AAGATACATT
481     GATGAGTTTG GACAAACCAC AACTAGAATG CAGTGAAAAA AATGCTTTAT TTGTGAAATT
541     TGTGATGCTA TTGCTTTATT TGTAACCATT ATAAGCTGCA ATAAACAAGT TAACAACAAC
601     AATTGCATTC ATTTTATGTT TCAGGTTCAG GGGGAGGTGT GGGAGGTTTT TTAAAGCAAG
661     TAAAACCTCT ACAAATGTGG TATGGCTGAT TATGATCAGT TATCTAGATC CGGTGGATCT
721     GAGTCCGGAC TTGTACAGCT CGTCCATGCC GAGAGTGATC CCGGCGGCGG TCACGAACTC
781     CAGCAGGACC ATGTGATCGC GCTTCTCGTT GGGGTCTTTG CTCAGGGCGG ACTGGGTGCT
841     CAGGTAGTGG TTGTCGGGCA GCAGCACGGG GCCGTCGCCG ATGGGGGTGT TCTGCTGGTA
901     GTGGTCGGCG AGCTGCACGC TGCCGTCCTC GATGTTGTGG CGGATCTTGA AGTTCACCTT
961     GATGCCGTTC TTCTGCTTGT CGGCCATGAT ATAGACGTTG TGGCTGTTGT AGTTGTACTC
1021    CAGCTTGTGC CCCAGGATGT TGCCGTCCTC CTTGAAGTCG ATGCCCTTCA GCTCGATGCG
1081    GTTCACCAGG GTGTCGCCCT CGAACTTCAC CTCGGCGCGG GTCTTGTAGT TGCCGTCGTC
1141    CTTGAAGAAG ATGGTGCGCT CCTGGACGTA GCCTTCGGGC ATGGCGGACT TGAAGAAGTC
1201    GTGCTGCTTC ATGTGGTCGG GGTAGCGGCT GAAGCACTGC ACGCCGTAGG TCAGGGTGGT
1261    CACGAGGGTG GGCCAGGGCA CGGGCAGCTT GCCGGTGGTG CAGATGAACT TCAGGGTCAG
```

1321 CTTGCCGTAG GTGGCATCGC CCTCGCCCTC GCCGGACACG CTGAACTTGT GGCCGTTTAC
1381 GTCGCCGTCC AGCTCGACCA GGATGGGCAC CACCCCGGTG AACAGCTCCT CGCCCTTGCT
1441 CACCATGGTG GCGACCGGTA GCGCTAGCGG ATCTGACGGT TCACTAAACC AGCTCTGCTT
1501 ATATAGACCT CCCACCGTAC ACGCCTACCG CCCATTGACG TCAATGGGAG TTTGTTTTGG
1561 CACCAAAATC AACGGGACTT TCCAAAATGT CGTAACAACT CCGCCCCATT GACGCAAATG
1621 GGCGGTAGGC GTGTACGGTG GGAGGTCTAT ATAAGCAGAG CTCTCTGGCT AACTAGAGAA
1681 CCCACTGCTT ACTGGCTTAT CGAAATTAAT ACGACTCACT ATAGGGAGAC CCAAGCTGGC
1741 TAGTTAAGCT TGGTACCGAG CTCGGATCCA CCATGGACAA AGACTGCGAA ATGAAGCGCA
1801 CCACCCTGGA TAGCCCTCTG GGCAAGCTGG AACTGTCTGG GTGCGAACAG GGCCTGCACC
1861 GTATCATCTT CCTGGGCAAA GGAACATCTG CCGCCGACGC CGTGGAAGTG CCTGCCCCAG
1921 CCGCCGTGCT GGGCGGACCA GAGCCACTGA TGCAGGCCAC CGCCTGGCTC AACGCCTACT
1981 TTCACCAGCC TGAGGCCATC GAGGAGTTCC CTGTGCCAGC CCTGCACCAC CCAGTGTTCC
2041 AGCAGGAGAG CTTTACCCGC CAGGTGCTGT GGAAACTGCT GAAAGTGGTG AAGTTCGGAG
2101 AGGTCATCAG CTACAGCCAC CTGGCCGCCC TGGCCGGCAA TCCCGCCGCC ACCGCCGCCG
2161 TGAAAACCGC CCTGAGCGGA AATCCCGTGC CCATTCTGAT CCCCTGCCAC CGGGTGGTGC
2221 AGGGCGACCT GGACGTGGGG GGCTACGAGG GCGGGCTCGC CGTGAAAGAG TGGCTGCTGG
2281 CCCACGAGGG CCACAGACTG GGCAAGCCTG GGCTGGGTCC TGCAGGCGGA GGCGCGCCAG
2341 GGTCTGGCGG CGGCAGTAAG GCAGAACGCA TGGGTTTCAC AGAGGTAACC CCAGTGACAG
2401 GGGCCAGTCT CAGAAGAACT ATGCTCCTCC TCTCAAGGTC CCCAGAAGCA CAGCCAAAGA
2461 CACTCCCTCT CACTGGCAGC ACCTTCCATG ACCAGATAGC CATGCTGAGC CACCGGTGCT
2521 TCAACACTCT GACTAACAGC TTCCAGCCCT CCTTGCTCGG CCGCAAGATT CTGGCCGCCA
2581 TCATTATGAA AAAAGACTCT GAGGACATGG GTGTCGTCGT CAGCTTGGGA ACAGGGAATC
2641 GCTGTGTAAA AGGAGATTCT CTCAGCCTAA AAGGAGAAAC TGTCAATGAC TGCCATGCAG
2701 AAATAATCTC CCGGAGAGGC TTCATCAGGT TTCTCTACAG TGAGTTAATG AAATACAACT
2761 CCCAGACTGC GAAGGATAGT ATATTTGAAC CTGCTAAGGG AGGAGAAAAG CTCCAAATAA
2821 AAAAGACTGT GTCATTCCAT CTGTATATCA GCACTGCTCC GTGTGGAGAT GGCGCCCTCT
2881 TTGACAAGTC CTGCAGCGAC CGTGCTATGG AAAGCACAGA ATCCCGCCAC TACCCTGTCT
2941 TCGAGAATCC CAAACAAGGA AAGCTCCGCA CCAAGGTGGA GAACGGACAA GGCACAATCC
3001 CTGTGGAATC CAGTGACATT GTGCCTACGT GGGATGGCAT TCGGCTCGGG GAGAGACTCC
3061 GTACCATGTC CTGTAGTGAC AAAATCCTAC GCTGGAACGT GCTGGGCCTG CAAGGGGCAC
3121 TGTTGACCCA CTTCCTGCAG CCCATTTATC TCAAATCTGT CACATTGGGT TACCTTTTCA
3181 GCCAAGGGCA TCTGACCCGT GCTATTTGCT GTCGTGTGAC AAGAGATGGG AGTGCATTTG
3241 AGGATGGACT ACGACATCCC TTTATTGTCA ACCACCCCAA GGTTGGCAGA GTCAGCATAT
3301 ATGATTCCAA AAGGCAATCC GGGAAGACTA AGGAGACAAG CGTCAACTGG TGTCTGGCTG
3361 ATGGCTATGA CCTGGAGATC CTGGACGGTA CCAGAGGCAC TGTGGATGGG CCACGGAATG
3421 AATTGTCCCG GGTCTCCAAA AAGAACATTT TTCTTCTATT TAAGAAGCTC TGCTCCTTCC
3481 GTTACCGCAG GGATCTACTG AGACTCTCCT ATGGTGAGGC CAAGAAAGCT GCCCGTGACT
3541 ACGAGACGGC CAAGAACTAC TTCAAAAAAG GCCTGAAGGA TATGGGCTAT GGGAACTGGA
3601 TTAGCAAACC CCAGGAGGAA AAGAACTTTT ATCTCTGCCC AGTATCTAGA GGGCCCTTCG
3661 AACAAAAACT CATCTCAGAA GAGGATCTGA ATATGCATAC CGGTCATCAT CACCATCACC
3721 ATTGAGTTGA AACCCGCTGA TCAGCCTCGA CTGTGCCTTC TAGTTGCCAG CCATCTGTTG
3781 TTTGCCCCTC CCCCGTGCCT TCCTTGACCC TGGAAGGTGC CACTCCCACT GTCCTTTCCT
3841 AATAAAATGA GGAAATTGCA TCGCATTGTC TGAGTAGGTG TCATTCTATT CTGGGGGGTG
3901 GGGTGGGGCA GGACAGCAAG GGGGAGGATT GGGAAGACAA TAGCAGGCAT GCTGGGGATG
3961 CGGTGGGCTC TATGGCTTCG AGAAGCTTTC TAGAGGATCT GGGCGTGGTT AAGGGTGGGA
4021 AAGAATATAT AAGGTGGGGG TCTTATGTAG TTTTGTATCT GTTTTGCAGC AGCCGCCGCC
4081 GCCATGAGCA CCAACTCGTT TGATGGAAGC ATTGTGAGCT CATATTTGAC AACGCGCATG
4141 CCCCCATGGG CCGGGGTGCG TCAGAATGTG ATGGGCTCCA GCATTGATGG TCGCCCCGTC
4201 CTGCCCGCAA ACTCTACTAC CTTGACCTAC GAGACCGTGT CTGGAACGCC GTTGGAGACT
4261 GCAGCCTCCG CCGCCGCTTC AGCCGCTGCA GCCACCGCCC GCGGGATTGT GACTGACTTT
4321 GCTTTCCTGA GCCCGCTTGC AAGCAGTGCA GCTTCCCGTT CATCCGCCCG CGATGACAAG
4381 TTGACGGCTC TTTTGGCACA ATTGGATTCT TTGACCCGGG AACTTAATGT CGTTTCTCAG
4441 CAGCTGTTGG ATCTGCGCCA GCAGGTTTCT GCCCTGAAGG CTTCCTCCCC TCCCAATGCG
4501 GTTTAAAACA TAAATAAAAA ACCAGACTCT GTTTGGATTT GGATCAAGCA AGTGTCTTGC
4561 TGTCTTTATT TAGGGGTTTT GCGCGCGCGG TAGGCCCGGG ACCAGCGGTC TCGGTCGTTG
4621 AGGGTCCTGT GTATTTTTTC CAGGACGTGG TAAAGGTGAC TCTGGATGTT CAGATACATG
4681 GGCATAAGCC CGTCTCTGGG GTGGAGGTAG CACCACTGCA GAGCTTCATG CTGCGGGGTG
4741 GTGTTGTAGA TGATCCAGTC GTAGCAGGAG CGCTGGGCGT GGTGCCTAAA AATGTCTTTC
4801 AGTAGCAAGC TGATTGCCAG GGCAGGCCCC TTGGTGTAAG TGTTTACAAA GCGGTTAAGC
4861 TGGGATGGGT GCATACGTGG GGATATGAGA TGCATCTTGG ACTGTATTTT TAGGTTGGCT
4921 ATGTTCCCAG CCATATCCCT CCGGGGATTC ATGTTGTGCA GAACCACCAG CACAGTGTAT

4981 CCGGTGCACT TGGGAAATTT GTCATGTAGC TTAGAAGGAA ATGCGTGGAA GAACTTGGAG
5041 ACGCCCTTGT GACCTCCAAG ATTTTCCATG CATTCGTCCA TAATGATGGC AATGGGCCCA
5101 CGGGCGGCGG CCTGGGCGAA GATATTTCTG GGATCACTAA CGTCATAGTT GTGTTCCAGG
5161 ATGAGATCGT CATAGGCCAT TTTTACAAAG CGCGGGCGGA GGGTGCCAGA CTGCGGTATA
5221 ATGGTTCCAT CCGGCCCAGG GGCGTAGTTA CCCTCACAGA TTTGCATTTC CCACGCTTTG
5281 AGTTCAGATG GGGGGATCAT GTCTACCTGC GGGGCGATGA AGAAAACGGT TTCCGGGGTA
5341 GGGGAGATCA GCTGGGAAGA AAGCAGGTTC CTGAGCAGCT GCGACTTACC GCAGCCGGTG
5401 GGCCCGTAAA TCACACCTAT TACCGGCTGC AACTGGTAGT TAAGAGAGCT GCAGCTGCCG
5461 TCATCCCTGA GCAGGGGGGC CACTTCGTTA AGCATGTCCC TGACTCGCAT GTTTTCCCTG
5521 ACCAAATCCG CCAGAAGGCG CTCGCCGCCC AGCGATAGCA GTTCTTGCAA GGAAGCAAAG
5581 TTTTTCAACG GTTTGAGACC GTCCGCCGTA GGCATGCTTT TGAGCGTTTG ACCAAGCAGT
5641 TCCAGGCGGT CCCACAGCTC GGTCACCTGC TCTACGGCAT CTCGATCCAG CATATCTCCT
5701 CGTTTCGCGG GTTGGGGCGG CTTTCGCTGT ACGGCAGTAG TCGGTGCTCG TCCAGACGGG
5761 CCAGGGTCAT GTCTTTCCAC GGGCGCAGGG TCCTCGTCAG CGTAGTCTGG GTCACGGTGA
5821 AGGGGTGCGC TCCGGGCTGC GCGCTGGCCA GGGTGCGCTT GAGGCTGGTC CTGCTGGTGC
5881 TGAAGCGCTG CCGGTCTTCG CCCTGCGCGT CGGCCAGGTA GCATTTGACC ATGGTGTCAT
5941 AGTCCAGCCC CTCCGCGGCG TGGCCCTTGG CGCGCAGCTT GCCCTTGGAG GAGGCGCCGC
6001 ACGAGGGGCA GTGCAGACTT TTGAGGGCGT AGAGCTTGGG CGCGAGAAAT ACCGATTCCG
6061 GGGAGTAGGC ATCCGCGCCG CAGGCCCCGC AGACGGTCTC GCATTCCACG AGCCAGGTGA
6121 GCTCTGGCCG TTCGGGGTCA AAAACCAGGT TTCCCCCATG CTTTTTGATG CGTTTCTTAC
6181 CTCTGGTTTC CATGAGCCGG TGTCCACGCT CGGTGACGAA AAGGCTGTCC GTGTCCCCGT
6241 ATACAGACTT GAGAGGGAGT TTAAACGAAT TCAATAGCTT GTTGCATGGG CGGCGATATA
6301 AAATGCAAGG TGCTGCTCAA AAAATCAGGC AAAGCCTCGC GCAAAAAAGA AAGCACATCG
6361 TAGTCATGCT CATGCAGATA AAGGCAGGTA AGCTCCGGAA CCACCACAGA AAAAGACACC
6421 ATTTTTCTCT CAAACATGTC TGCGGGTTTC TGCATAAACA CAAAATAAAA TAACAAAAAA
6481 ACATTTAAAC ATTAGAAGCC TGTCTTACAA CAGGAAAAAC AACCCTTATA AGCATAAGAC
6541 GGACTACGGC CATGCCGGCG TGACCGTAAA AAAACTGGTC ACCGTGATTA AAAAGCACCA
6601 CCGACAGCTC CTCGGTCATG TCCGGAGTCA TAATGTAAGA CTCGGTAAAC ACATCAGGTT
6661 GATTCACATC GGTCAGTGCT AAAAAGCGAC CGAAATAGCC CGGGGGAATA CATACCCGCA
6721 GGCGTAGAGA CAACATTACA GCCCCCATAG GAGGTATAAC AAAATTAATA GGAGAGAAAA
6781 ACACATAAAC ACCTGAAAAA CCCTCCTGCC TAGGCAAAAT AGCACCCTCC CGCTCCAGAA
6841 CAACATACAG CGCTTCCACA GCGGCAGCCA TAACAGTCAG CCTTACCAGT AAAAAAGAAA
6901 ACCTATTAAA AAAACACCAC TCGACACGGC ACCAGCTCAA TCAGTCACAG TGTAAAAAAG
6961 GGCCAAGTGC AGAGCGAGTA TATATAGGAC TAAAAAATGA CGTAACGGTT AAAGTCCACA
7021 AAAAACACCC AGAAAACCGC ACGCGAACCT ACGCCCAGAA ACGAAAGCCA AAAAACCCAC
7081 AACTTCCTCA AATCGTCACT TCCGTTTTCC CACGTTACGT CACTTCCCAT TTTAAGAAAA
7141 CTACAATTCC CAACACATAC AAGTTACTCC GCCCTAAAAC CTACGTCACC CGCCCCGTTC
7201 CCACGCCCCG CGCCACGTCA CAAACTCCAC CCCCTCATTA TCATATTGGC TTCAATCCAA
7261 AATAAGGTAT ATTATTGATG ATGTTAATTA ACATGCATGG ATCCATATGC GGTGTGAAAT
7321 ACCGCACAGA TGCGTAAGGA GAAAATACCG CATCAGGCGC TCTTCCGCTT CCTCGCTCAC
7381 TGACTCGCTG CGCTCGGTCG TTCGGCTGCG GCGAGCGGTA TCAGCTCACT CAAAGGCGGT
7441 AATACGGTTA TCCACAGAAT CAGGGGATAA CGCAGGAAAG AACATGTGAG CAAAAGGCCA
7501 GCAAAAGGCC AGGAACCGTA AAAAGGCCGC GTTGCTGGCG TTTTTCCATA GGCTCCGCCC
7561 CCCTGACGAG CATCACAAAA ATCGACGCTC AAGTCAGAGG TGGCGAAACC CGACAGGACT
7621 ATAAAGATAC CAGGCGTTTC CCCCTGGAAG CTCCCTCGTG CGCTCTCCTG TTCCGACCCT
7681 GCCGCTTACC GGATACCTGT CCGCCTTTCT CCCTTCGGGA AGCGTGGCGC TTTCTCATAG
7741 CTCACGCTGT AGGTATCTCA GTTCGGTGTA GGTCGTTCGC TCCAAGCTGG GCTGTGTGCA
7801 CGAACCCCCC GTTCAGCCCG ACCGCTGCGC CTTATCCGGT AACTATCGTC TTGAGTCCAA
7861 CCCGGTAAGA CACGACTTAT CGCCACTGGC AGCAGCCACT GGTAACAGGA TTAGCAGAGC
7921 GAGGTATGTA GGCGGTGCTA CAGAGTTCTT GAAGTGGTGG CCTAACTACG GCTACACTAG
7981 AAGGACAGTA TTTGGTATCT GCGCTCTGCT GAAGCCAGTT ACCTTCGGAA AAAGAGTTGG
8041 TAGCTCTTGA TCCGGCAAAC AAACCACCGC TGGTAGCGGT GGTTTTTTTG TTTGCAAGCA
8101 GCAGATTACG CGCAGAAAAA AAGGATCTCA AGAAGATCCT TTGATCTTTT CTACGGGGTC
8161 TGACGCTCAG TGGAACGAAA ACTCACGTTA AGGGATTTTG GTCATGAGAT TATCAAAAAG
8221 GATCTTCACC TAGATCCTTT TAAATTAAAA ATGAAGTTTT AAATCAATCT AAAGTATATA
8281 TGAGTAAACT TGGTCTGACA GTTACCAATG CTTAATCAGT GAGGCACCTA TCTCAGCGAT
8341 CTGTCTATTT CGTTCATCCA TAGTTGCCTG ACTCCCCGTC GTGTAGATAA CTACGATACG
8401 GGAGGGCTTA CCATCTGGCC CCAGTGCTGC AATGATACCG CGAGACCCAC GCTCACCGGC
8461 TCCAGATTTA TCAGCAATAA ACCAGCCAGC CGGAAGGGCC GAGCGCAGAA GTGGTCCTGC
8521 AACTTTATCC GCCTCCATCC AGTCTATTAA TTGTTGCCGG GAAGCTAGAG TAAGTAGTTC
8581 GCCAGTTAAT AGTTTGCGCA ACGTTGTTGC CATTGCTGCA GCCATGAGAT TATCAAAAAG

8641 GATCTTCACC TAGATCCTTT TCACGTAGAA AGCCAGTCCG CAGAAACGGT GCTGACCCCG
8701 GATGAATGTC AGCTACTGGG CTATCTGGAC AAGGGAAAAC GCAAGCGCAA AGAGAAAGCA
8761 GGTAGCTTGC AGTGGGCTTA CATGGCGATA GCTAGACTGG GCGGTTTTAT GGACAGCAAG
8821 CGAACCGGAA TTGCCAGCTG GGGCGCCCTC TGGTAAGGTT GGGAAGCCCT GCAAAGTAAA
8881 CTGGATGGCT TTCTTGCCGC CAAGGATCTG ATGGCGCAGG GGATCAAGCT CTGATCAAGA
8941 GACAGGATGA GGATCGTTTC GCATGATTGA ACAAGATGGA TTGCACGCAG GTTCTCCGGC
9001 CGCTTGGGTG GAGAGGCTAT TCGGCTATGA CTGGGCACAA CAGACAATCG GCTGCTCTGA
9061 TGCCGCCGTG TTCCGGCTGT CAGCGCAGGG GCGCCCGGTT CTTTTTGTCA AGACCGACCT
9121 GTCCGGTGCC CTGAATGAAC TGCAAGACGA GGCAGCGCGG CTATCGTGGC TGGCCACGAC
9181 GGGCGTTCCT TGCGCAGCTG TGCTCGACGT TGTCACTGAA GCGGGAAGGG ACTGGCTGCT
9241 ATTGGGCGAA GTGCCGGGGC AGGATCTCCT GTCATCTCAC CTTGCTCCTG CCGAGAAAGT
9301 ATCCATCATG GCTGATGCAA TGCGGCGGCT GCATACGCTT GATCCGGCTA CCTGCCCATT
9361 CGACCACCAA GCGAAACATC GCATCGAGCG AGCACGTACT CGGATGGAAG CCGGTCTTGT
9421 CGATCAGGAT GATCTGGACG AAGAGCATCA GGGGCTCGCG CCAGCCGAAC TGTTCGCCAG
9481 GCTCAAGGCG AGCATGCCCG ACGGCGAGGA TCTCGTCGTG ACCCATGGCG ATGCCTGCTT
9541 GCCGAATATC ATGGTGGAAA ATGGCCGCTT TTCTGGATTC ATCGACTGTG GCCGGCTGGG
9601 TGTGGCGGAC CGCTATCAGG ACATAGCGTT GGCTACCCGT GATATTGCTG AAGAGCTTGG
9661 CGGCGAATGG GCTGACCGCT TCCTCGTGCT TTACGGTATC GCCGCTCCCG ATTCGCAGCG
9721 CATCGCCTTC TATCGCCTTC TTGACGAGTT CTTCTGAATT TTGTTAAAAT TTTTGTTAAA
9781 TCAGCTCATT TTTTAACCAA TAGGCCGAAA TCGGCAAAAT CCCTTATAAA TCAAAAGAAT
9841 AGACCGAGAT AGGGTTGAGT GTTGTTCCAG TTTGGAACAA GAGTCCACTA TTAAAGAACG
9901 TGGACTCCAA CGTCAAAGGG CGAAAAACCG TCTATCAGGG CGATGGCCCA CTACGTGAAC
9961 CATCACCCTA ATCAAGTTTT TTGGGGTCGA GGTGCCGTAA AGCACTAAAT CGGAACCCTA
10021 AAGGGAGCCC CCGATTTAGA GCTTGACGGG GAAAGCCGGC GAACGTGGCG AGAAAGGAAG
10081 GGAAGAAAGC GAAAGGAGCG GGCGCTAGGG CGCTGGCAAG TGTAGCGGTC ACGCTGCGCG
10141 TAACCACCAC ACCCGCCGCG CTTAATGCGC CGCTACAGGG CGCGTCCATT CGCCATTCAG
10201 GATCGAATTA ATTCTTAATT AACATCATCA ATAATA

Plasmid-map and sequence of EF1A_GFP_SA1Q_pLentiPuro (pTS1025). E>Q site is highlighted in cyan:

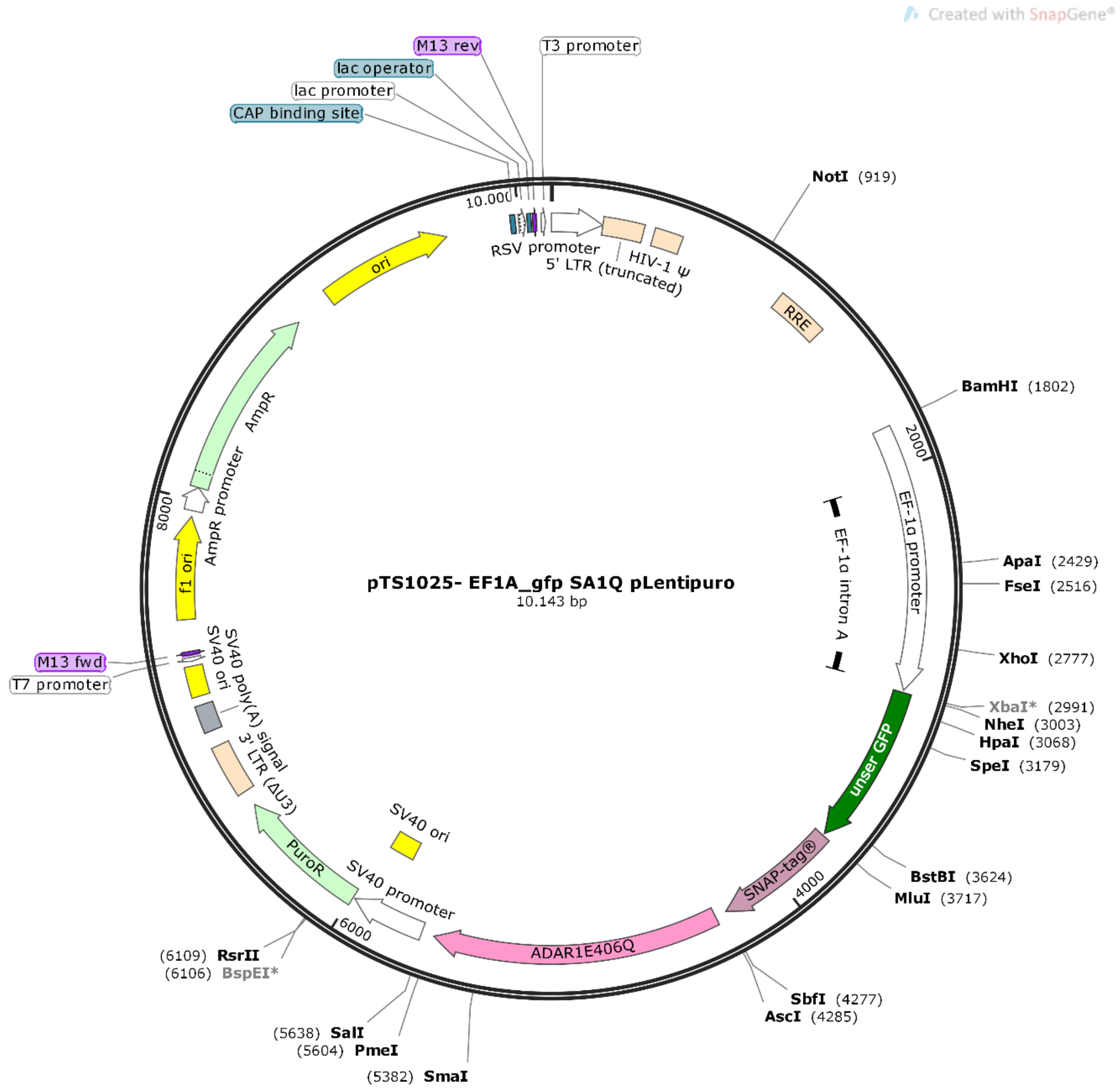


```
1          ATGTAGTCTT ATGCAATACT CTTGTAGTCT TGCAACATGG TAACGATGAG TTAGCAACAT
61         GCCTTACAAG GAGAGAAAAA GCACCGTGCA TGCCGATTGG TGGAAGTAAG GTGGTACGAT
121        CGTGCCTTAT TAGGAAGGCA ACAGACGGGT CTGACATGGA TTGGACGAAC CACTGAATTG
181        CCGCATTGCA GAGATATTGT ATTTAAGTGC CTAGCTCGAT ACATAAACGG GTCTCTCTGG
241        TTAGACCAGA TCTGAGCCTG GGAGCTCTCT GGCTAACTAG GGAACCCACT GCTTAAGCCT
301        CAATAAAGCT TGCCTTGAGT GCTTCAAGTA GTGTGTGCCC GTCTGTTGTG TGACTCTGGT
361        AACTAGAGAT CCCTCAGACC CTTTTAGTCA GTGTGGAAAA TCTCTAGCAG TGGCGCCCGA
421        ACAGGGACTT GAAAGCGAAA GGGAAACCAG AGGAGCTCTC TCGACGCAGG ACTCGGCTTG
481        CTGAAGCGCG CACGGCAAGA GGCGAGGGGC GGCGACTGGT GAGTACGCCA AAAATTTTGA
541        CTAGCGGAGG CTAGAAGGAG AGAGATGGGT GCGAGAGCGT CAGTATTAAG CGGGGGAGAA
601        TTAGATCGCG ATGGGAAAAA ATTCGGTTAA GGCCAGGGGG AAAGAAAAAA TATAAATTAA
661        AACATATAGT ATGGGCAAGC AGGGAGCTAG AACGATTCGC AGTTAATCCT GGCCTGTTAG
721        AAACATCAGA AGGCTGTAGA CAAATACTGG GACAGCTACA ACCATCCCTT CAGACAGGAT
781        CAGAAGAACT TAGATCATTA TATAATACAG TAGCAACCCT CTATTGTGTG CATCAAAGGA
841        TAGAGATAAA AGACACCAAG GAAGCTTTAG ACAAGATAGA GGAAGAGCAA AACAAAAGTA
901        AGACCACCGC ACAGCAAGCG GCCGCTGATC TTCAGACCTG GAGGAGGAGA TATGAGGGAC
961        AATTGGAGAA GTGAATTATA TAAATATAAA GTAGTAAAAA TTGAACCATT AGGAGTAGCA
```

1021 CCCACCAAGG CAAAGAGAAG AGTGGTGCAG AGAGAAAAAA GAGCAGTGGG AATAGGAGCT
1081 TTGTTCCTTG GGTTCTTGGG AGCAGCAGGA AGCACTATGG GCGCAGCGTC AATGACGCTG
1141 ACGGTACAGG CCAGACAATT ATTGTCTGGT ATAGTGCAGC AGCAGAACAA TTTGCTGAGG
1201 GCTATTGAGG CGCAACAGCA TCTGTTGCAA CTCACAGTCT GGGGCATCAA GCAGCTCCAG
1261 GCAAGAATCC TGGCTGTGGA AAGATACCTA AAGGATCAAC AGCTCCTGGG GATTTGGGGT
1321 TGCTCTGGAA AACTCATTTG CACCACTGCT GTGCCTTGGA ATGCTAGTTG GAGTAATAAA
1381 TCTCTGGAAC AGATTTGGAA TCACACGACC TGGATGGAGT GGGACAGAGA AATTAACAAT
1441 TACACAAGCT TAATACACTC CTTAATTGAA GAATCGCAAA ACCAGCAAGA AAAGAATGAA
1501 CAAGAATTAT TGGAATTAGA TAAATGGGCA AGTTTGTGGA ATTGGTTTAA CATAACAAAT
1561 TGGCTGTGGT ATATAAAATT ATTCATAATG ATAGTAGGAG GCTTGGTAGG TTTAAGAATA
1621 GTTTTTGCTG TACTTTCTAT AGTGAATAGA GTTAGGCAGG GATATTCACC ATTATCGTTT
1681 CAGACCCACC TCCCAACCCC GAGGGGACCC GACAGGCCCG AAGGAATAGA AGAAGAAGGT
1741 GGAGAGAGAG ACAGAGACAG ATCCATTCGA TTAGTGAACG GATCTCGACG GTATCGCTAG
1801 TGGATCCGTG AGGCTCCGGT GCCCGTCAGT GGGCAGAGCG CACATCGCCC ACAGTCCCCG
1861 AGAAGTTGGG GGGAGGGGTC GGCAATTGAA CCGGTGCCTA GAGAAGGTGG CGCGGGGTAA
1921 ACTGGGAAAG TGATGTCGTG TACTGGCTCC GCCTTTTTCC CGAGGGTGGG GGAGAACCGT
1981 ATATAAGTGC AGTAGTCGCC GTGAACGTTC TTTTTCGCAA CGGGTTTGCC GCCAGAACAC
2041 AGGTAAGTGC CGTGTGTGGT TCCCGCGGGC CTGGCCTCTT TACGGGTTAT GGCCCTTGCG
2101 TGCCTTGAAT TACTTCCACC TGGCTGCAGT ACGTGATTCT TGATCCCGAG CTTCGGGTTG
2161 GAAGTGGGTG GGAGAGTTCG AGGCCTTGCG CTTAAGGAGC CCCTTCGCCT CGTGCTTGAG
2221 TTGAGGCCTG GCCTGGGCGC TGGGGCCGCC GCGTGCGAAT CTGGTGGCAC CTTCGCGCCT
2281 GTCTCGCTGC TTTCGATAAG TCTCTAGCCA TTTAAAATTT TTGATGACCT GCTGCGACGC
2341 TTTTTTTCTG GCAAGATAGT CTTGTAAATG CGGGCCAAGA TCTGCACACT GGTATTTCGG
2401 TTTTTGGGGC CGCGGGCGGC GACGGGGCCC GTGCGTCCCA GCGCACATGT TCGGCGAGGC
2461 GGGGCCTGCG AGCGCGGCCA CCGAGAATCG GACGGGGGTA GTCTCAAGCT GGCCGGCCTG
2521 CTCTGGTGCC TGGCCTCGCG CCGCCGTGTA TCGCCCCGCC CTGGGCGGCA AGGCTGGCCC
2581 GGTCGGCACC AGTTGCGTGA GCGGAAAGAT GGCCGCTTCC CGGCCCTGCT GCAGGGAGCT
2641 CAAAATGGAG GACGCGGCGC TCGGGAGAGC GGGCGGGTGA GTCACCCACA CAAAGGAAAA
2701 GGGCCTTTCC GTCCTCAGCC GTCGCTTCAT GTGACTCCAC GGAGTACCGG GCGCCGTCCA
2761 GGCACCTCGA TTAGTTCTCG AGCTTTTGGA GTACGTCGTC TTTAGGTTGG GGGGAGGGGT
2821 TTTATGCGAT GGAGTTTCCC CACACTGAGT GGGTGGAGAC TGAAGTTAGG CCAGCTTGGC
2881 ACTTGATGTA ATTCTCCTTG GAATTTGCCC TTTTTGAGTT TGGATCTTGG TTCATTCTCA
2941 AGCCTCAGAC AGTGGTTCAA AGTTTTTTTC TTCCATTTCA GGTGTCGTGA TCTAGAACCA
3001 TGGCTAGCAA AGGAGAAGAA CTCTTCACTG GAGTTGTCCC AATTCTTGTT GAATTAGATG
3061 GTGATGTTAA CGGCCACAAG TTCTCTGTCA GTGGAGAGGG TGAAGGTGAT GCAACATACG
3121 GAAAACTTAC CCTGAAGTTC ATCTGCACTA CTGGCAAACT GCCTGTTCCG TGGCCGACAC
3181 TAGTGACGAC GCTCTGCTAT GGCGTCCAGT GCTTTTCAAG ATACCCGGAT CACATGAAAC
3241 GGCATGACTT TTTCAAGAGT GCCATGCCCG AAGGTTATGT ACAGGAAAGG ACCATCTTCT
3301 TCAAAGATGA CGGCAACTAC AAGACACGTG CTGAAGTCAA GTTTGAAGGT GATACCCTTG
3361 TTAATAGAAT CGAGTTAAAA GGTATTGACT TCAAGGAAGA TGGCAACATT CTGGGACACA
3421 AATTGGAATA CAACTATAAC TCACACAATG TATACATCAT GGCAGACAAA CAAAAGAATG
3481 GAATCAAAGT GAACTTCAAG ACCCGCCACA ACATTGAAGA TGGAAGCGTT CAACTAGCAG
3541 ACCATTATCA ACAAAATACT CCAATTGGCG ATGGCCCTGT CCTTTTACCA GACAACCATT
3601 ACCTGTCCAC ACAATCTGCC CTTTCGAAAG ATCCCAACGA AAAGAGAGAC CACATGGTCC
3661 TTCTTGAGTT TGTAACAGCT GCTGGGATTA CACATGGCAT GGATGAACTA TACAAAACGC
3721 GTACCATGGA CAAAGACTGC GAAATGAAGC GCACCACCCT GGATAGCCCT CTGGGCAAGC
3781 TGGAACTGTC TGGGTGCGAA CAGGGCCTGC ACCGTATCAT CTTCCTGGGC AAAGGAACAT
3841 CTGCCGCCGA CGCCGTGGAA GTGCCTGCCC CAGCCGCCGT GCTGGGCGGA CCAGAGCCAC
3901 TGATGCAGGC CACCGCCTGG CTCAACGCCT ACTTTCACCA GCCTGAGGCC ATCGAGGAGT
3961 TCCCTGTGCC AGCCCTGCAC CACCCAGTGT TCCAGCAGGA GAGCTTTACC CGCCAGGTGC
4021 TGTGGAAACT GCTGAAAGTG GTGAAGTTCG GAGAGGTCAT CAGCTACAGC CACCTGGCCG
4081 CCCTGGCCGG CAATCCCGCC GCCACCGCCG CCGTGAAAAC CGCCCTGAGC GGAAATCCCG
4141 TGCCCATTCT GATCCCCTGC CACCGGGTGG TGCAGGGCGA CCTGGACGTG GGGGGCTACG
4201 AGGGCGGGCT CGCCGTGAAA GAGTGGCTGC TGGCCCACGA GGGCCACAGA CTGGGCAAGC
4261 CTGGGCTGGG TCCTGCAGGC GGAGGCGCGC CAGGGTCTGG CGGCGGCAGT AAGGCAGAAC
4321 GCATGGGTTT CACAGAGGTA ACCCCAGTGA CAGGGGCCAG TCTCAGAAGA ACTATGCTCC
4381 TCCTCTCAAG GTCCCCAGAA GCACAGCCAA AGACACTCCC TCTCACTGGC AGCACCTTCC
4441 ATGACCAGAT AGCCATGCTG AGCCACCGGT GCTTCAACAC TCTGACTAAC AGCTTCCAGC
4501 CCTCCTTGCT CGGCCGCAAG ATTCTGGCCG CCATCATTAT GAAAAAAGAC TCTGAGGACA
4561 TGGGTGTCGT CGTCAGCTTG GGAACAGGGA ATCGCTGTGT AAAAGGAGAT TCTCTCAGCC
4621 TAAAAGGAGA AACTGTCAAT GACTGCCATG CAGAAATAAT CTCCCGGAGA GGCTTCATCA

4681 GGTTTCTCTA CAGTGAGTTA ATGAAATACA ACTCCCAGAC TGCGAAGGAT AGTATATTTG
4741 AACCTGCTAA GGGAGGAGAA AAGCTCCAAA TAAAAAAGAC TGTGTCATTC CATCTGTATA
4801 TCAGCACTGC TCCGTGTGGA GATGGCGCCC TCTTTGACAA GTCCTGCAGC GACCGTGCTA
4861 TGGAAAGCAC AGAATCCCGC CACTACCCTG TCTTCGAGAA TCCCAAACAA GGAAAGCTCC
4921 GCACCAAGGT GGAGAACGGA CAAGGCACAA TCCCTGTGGA ATCCAGTGAC ATTGTGCCTA
4981 CGTGGGATGG CATTCGGCTC GGGGAGAGAC TCCGTACCAT GTCCTGTAGT GACAAAATCC
5041 TACGCTGGAA CGTGCTGGGC CTGCAAGGGG CACTGTTGAC CCACTTCCTG CAGCCCATTT
5101 ATCTCAAATC TGTCACATTG GGTTACCTTT TCAGCCAAGG GCATCTGACC CGTGCTATTT
5161 GCTGTCGTGT GACAAGAGAT GGGAGTGCAT TTGAGGATGG ACTACGACAT CCCTTTATTG
5221 TCAACCACCC CAAGGTTGGC AGAGTCAGCA TATATGATTC CAAAAGGCAA TCCGGGAAGA
5281 CTAAGGAGAC AAGCGTCAAC TGGTGTCTGG CTGATGGCTA TGACCTGGAG ATCCTGGACG
5341 GTACCAGAGG CACTGTGGAT GGGCCACGGA ATGAATTGTC CCGGGTCTCC AAAAAGAACA
5401 TTTTTCTTCT ATTTAAGAAG CTCTGCTCCT TCCGTTACCG CAGGGATCTA CTGAGACTCT
5461 CCTATGGTGA GGCCAAGAAA GCTGCCCGTG ACTACGAGAC GGCCAAGAAC TACTTCAAAA
5521 AAGGCCTGAA GGATATGGGC TATGGGAACT GGATTAGCAA ACCCCAGGAG GAAAAGAACT
5581 TTTATCTCTG CCCAGTATAA GTTTAAACCT GCTGATCAGC CTCGACTGTG CCTTCTAGTC
5641 GACCCTGTGG AATGTGTGTC AGTTAGGGTG TGGAAAGTCC CCAGGCTCCC CAGCAGGCAG
5701 AAGTATGCAA AGCATGCATC TCAATTAGTC AGCAACCAGG TGTGGAAAGT CCCCAGGCTC
5761 CCCAGCAGGC AGAAGTATGC AAAGCATGCA TCTCAATTAG TCAGCAACCA TAGTCCCGCC
5821 CCTAACTCCG CCCATCCCGC CCCTAACTCC GCCCAGTTCC GCCCATTCTC CGCCCCATGG
5881 CTGACTAATT TTTTTTATTT ATGCAGAGGC CGAGGCCGCC TCGGCCTCTG AGCTATTCCA
5941 GAAGTAGTGA GGAGGCTTTT TTGGAGGCCT AGGCTTTTGC AAAAAGCTTA CCATGACCGA
6001 GTACAAGCCC ACGGTGCGCC TCGCCACCCG CGACGACGTC CCCAGGGCCG TACGCACCCT
6061 CGCCGCCGCG TTCGCCGACT ACCCCGCCAC GCGCCACACC GTCGATCCGG ACCGCCACAT
6121 CGAGCGGGTC ACCGAGCTGC AAGAACTCTT CCTCACGCGC GTCGGGCTCG ACATCGGCAA
6181 GGTGTGGGTC GCGGACGACG GCGCCGCGGT GGCGGTCTGG ACCACGCCGG AGAGCGTCGA
6241 AGCGGGGGCG GTGTTCGCCG AGATCGGCCC GCGCATGGCC GAGTTGAGCG GTTCCCGGCT
6301 GGCCGCGCAG CAACAGATGG AAGGCCTCCT GGCGCCGCAC CGGCCCAAGG AGCCCGCGTG
6361 GTTCCTGGCC ACCGTCGGCG TCTCGCCCGA CCACCAGGGC AAGGGTCTGG GCAGCGCCGT
6421 CGTGCTCCCC GGAGTGGAGG CGGCCGAGCG CGCCGGGGTG CCCGCCTTCC TGGAGACCTC
6481 CGCGCCCCGC AACCTCCCCT TCTACGAGCG GCTCGGCTTC ACCGTCACCG CCGACGTCGA
6541 GTGCCCGAAG GACCGCGCGA CCTGGTGCAT GACCCGCAAG CCCGGTGCCT GAGGTACCTT
6601 TAAGACCAAT GACTTACAAG GCAGCTGTAG ATCTTAGCCA CTTTTTAAAA GAAAAGGGGG
6661 GACTGGAAGG GCTAATTCAC TCCCAACGAA GACAAGATCT GCTTTTTGCT TGTACTGGGT
6721 CTCTCTGGTT AGACCAGATC TGAGCCTGGG AGCTCTCTGG CTAACTAGGG AACCCACTGC
6781 TTAAGCCTCA ATAAAGCTTG CCTTGAGTGC TTCAAGTAGT GTGTGCCCGT CTGTTGTGTG
6841 ACTCTGGTAA CTAGAGATCC CTCAGACCCT TTTAGTCAGT GTGGAAAATC TCTAGCAGTA
6901 GTAGTTCATG TCATCTTATT ATTCAGTATT TATAACTTGC AAAGAAATGA ATATCAGAGA
6961 GTGAGAGGAA CTTGTTTATT GCAGCTTATA ATGGTTACAA ATAAAGCAAT AGCATCACAA
7021 ATTTCACAAA TAAAGCATTT TTTTCACTGC ATTCTAGTTG TGGTTTGTCC AAACTCATCA
7081 ATGTATCTTA TCATGTCTGG CTCTAGCTAT CCCGCCCCTA ACTCCGCCCA TCCCGCCCCT
7141 AACTCCGCCC AGTTCCGCCC ATTCTCCGCC CCATGGCTGA CTAATTTTTT TTATTTATGC
7201 AGAGGCCGAG GCCGCCTCGG CCTCTGAGCT ATTCCAGAAG TAGTGAGGAG GCTTTTTTGG
7261 AGGCCTAGGG ACGTACCCAA TTCGCCCTAT AGTGAGTCGT ATTACGCGCG CTCACTGGCC
7321 GTCGTTTTAC AACGTCGTGA CTGGGAAAAC CCTGGCGTTA CCCAACTTAA TCGCCTTGCA
7381 GCACATCCCC CTTTCGCCAG CTGGCGTAAT AGCGAAGAGG CCCGCACCGA TCGCCCTTCC
7441 CAACAGTTGC GCAGCCTGAA TGGCGAATGG GACGCGCCCT GTAGCGGCGC ATTAAGCGCG
7501 GCGGGTGTGG TGGTTACGCG CAGCGTGACC GCTACACTTG CCAGCGCCCT AGCGCCCGCT
7561 CCTTTCGCTT TCTTCCCTTC CTTTCTCGCC ACGTTCGCCG GCTTTCCCCG TCAAGCTCTA
7621 AATCGGGGGC TCCCTTTAGG GTTCCGATTT AGTGCTTTAC GGCACCTCGA CCCCAAAAAA
7681 CTTGATTAGG GTGATGGTTC ACGTAGTGGG CCATCGCCCT GATAGACGGT TTTTCGCCCT
7741 TTGACGTTGG AGTCCACGTT CTTTAATAGT GGACTCTTGT TCCAAACTGG AACAACACTC
7801 AACCCTATCT CGGTCTATTC TTTTGATTTA TAAGGGATTT TGCCGATTTC GGCCTATTGG
7861 TTAAAAAATG AGCTGATTTA ACAAAAATTT AACGCGAATT TTAACAAAAT ATTAACGCTT
7921 ACAATTTAGG TGGCACTTTT CGGGGAAATG TGCGCGGAAC CCCTATTTGT TTATTTTTCT
7981 AAATACATTC AAATATGTAT CCGCTCATGA GACAATAACC CTGATAAATG CTTCAATAAT
8041 ATTGAAAAAG GAAGAGTATG AGTATTCAAC ATTTCCGTGT CGCCCTTATT CCCTTTTTTG
8101 CGGCATTTTG CCTTCCTGTT TTTGCTCACC CAGAAACGCT GGTGAAAGTA AAAGATGCTG
8161 AAGATCAGTT GGGTGCACGA GTGGGTTACA TCGAACTGGA TCTCAACAGC GGTAAGATCC
8221 TTGAGAGTTT TCGCCCCGAA GAACGTTTTC CAATGATGAG CACTTTTAAA GTTCTGCTAT
8281 GTGGCGCGGT ATTATCCCGT ATTGACGCCG GGCAAGAGCA ACTCGGTCGC CGCATACACT

8341 ATTCTCAGAA TGACTTGGTT GAGTACTCAC CAGTCACAGA AAAGCATCTT ACGGATGGCA
8401 TGACAGTAAG AGAATTATGC AGTGCTGCCA TAACCATGAG TGATAACACT GCGGCCAACT
8461 TACTTCTGAC AACGATCGGA GGACCGAAGG AGCTAACCGC TTTTTTGCAC AACATGGGGG
8521 ATCATGTAAC TCGCCTTGAT CGTTGGGAAC CGGAGCTGAA TGAAGCCATA CCAAACGACG
8581 AGCGTGACAC CACGATGCCT GTAGCAATGG CAACAACGTT GCGCAAACTA TTAACTGGCG
8641 AACTACTTAC TCTAGCTTCC CGGCAACAAT TAATAGACTG GATGGAGGCG GATAAAGTTG
8701 CAGGACCACT TCTGCGCTCG GCCCTTCCGG CTGGCTGGTT TATTGCTGAT AAATCTGGAG
8761 CCGGTGAGCG TGGGTCTCGC GGTATCATTG CAGCACTGGG GCCAGATGGT AAGCCCTCCC
8821 GTATCGTAGT TATCTACACG ACGGGGAGTC AGGCAACTAT GGATGAACGA AATAGACAGA
8881 TCGCTGAGAT AGGTGCCTCA CTGATTAAGC ATTGGTAACT GTCAGACCAA GTTTACTCAT
8941 ATATACTTTA GATTGATTTA AAACTTCATT TTTAATTTAA AAGGATCTAG GTGAAGATCC
9001 TTTTTGATAA TCTCATGACC AAAATCCCTT AACGTGAGTT TTCGTTCCAC TGAGCGTCAG
9061 ACCCCGTAGA AAAGATCAAA GGATCTTCTT GAGATCCTTT TTTTCTGCGC GTAATCTGCT
9121 GCTTGCAAAC AAAAAAACCA CCGCTACCAG CGGTGGTTTG TTTGCCGGAT CAAGAGCTAC
9181 CAACTCTTTT TCCGAAGGTA ACTGGCTTCA GCAGAGCGCA GATACCAAAT ACTGTTCTTC
9241 TAGTGTAGCC GTAGTTAGGC CACCACTTCA AGAACTCTGT AGCACCGCCT ACATACCTCG
9301 CTCTGCTAAT CCTGTTACCA GTGGCTGCTG CCAGTGGCGA TAAGTCGTGT CTTACCGGGT
9361 TGGACTCAAG ACGATAGTTA CCGGATAAGG CGCAGCGGTC GGGCTGAACG GGGGGTTCGT
9421 GCACACAGCC CAGCTTGGAG CGAACGACCT ACACCGAACT GAGATACCTA CAGCGTGAGC
9481 TATGAGAAAG CGCCACGCTT CCCGAAGGGA GAAAGGCGGA CAGGTATCCG GTAAGCGGCA
9541 GGGTCGGAAC AGGAGAGCGC ACGAGGGAGC TTCCAGGGGG AAACGCCTGG TATCTTTATA
9601 GTCCTGTCGG GTTTCGCCAC CTCTGACTTG AGCGTCGATT TTTGTGATGC TCGTCAGGGG
9661 GGCGGAGCCT ATGGAAAAAC GCCAGCAACG CGGCCTTTTT ACGGTTCCTG GCCTTTTGCT
9721 GGCCTTTTGC TCACATGTTC TTTCCTGCGT TATCCCCTGA TTCTGTGGAT AACCGTATTA
9781 CCGCCTTTGA GTGAGCTGAT ACCGCTCGCC GCAGCCGAAC GACCGAGCGC AGCGAGTCAG
9841 TGAGCGAGGA AGCGGAAGAG CGCCCAATAC GCAAACCGCC TCTCCCCGCG CGTTGGCCGA
9901 TTCATTAATG CAGCTGGCAC GACAGGTTTC CCGACTGGAA AGCGGGCAGT GAGCGCAACG
9961 CAATTAATGT GAGTTAGCTC ACTCATTAGG CACCCCAGGC TTTACACTTT ATGCTTCCGG
10021 CTCGTATGTT GTGTGGAATT GTGAGCGGAT AACAATTTCA CACAGGAAAC AGCTATGACC
10081 ATGATTACGC CAAGCGCGCA ATTAACCCTC ACTAAAGGGA ACAAAAGCTG GAGCTGCAAG
10141 CTT

Plasmid-map and sequence of EF1A_GFP_SA1_pLentiPuro (pTS1036):

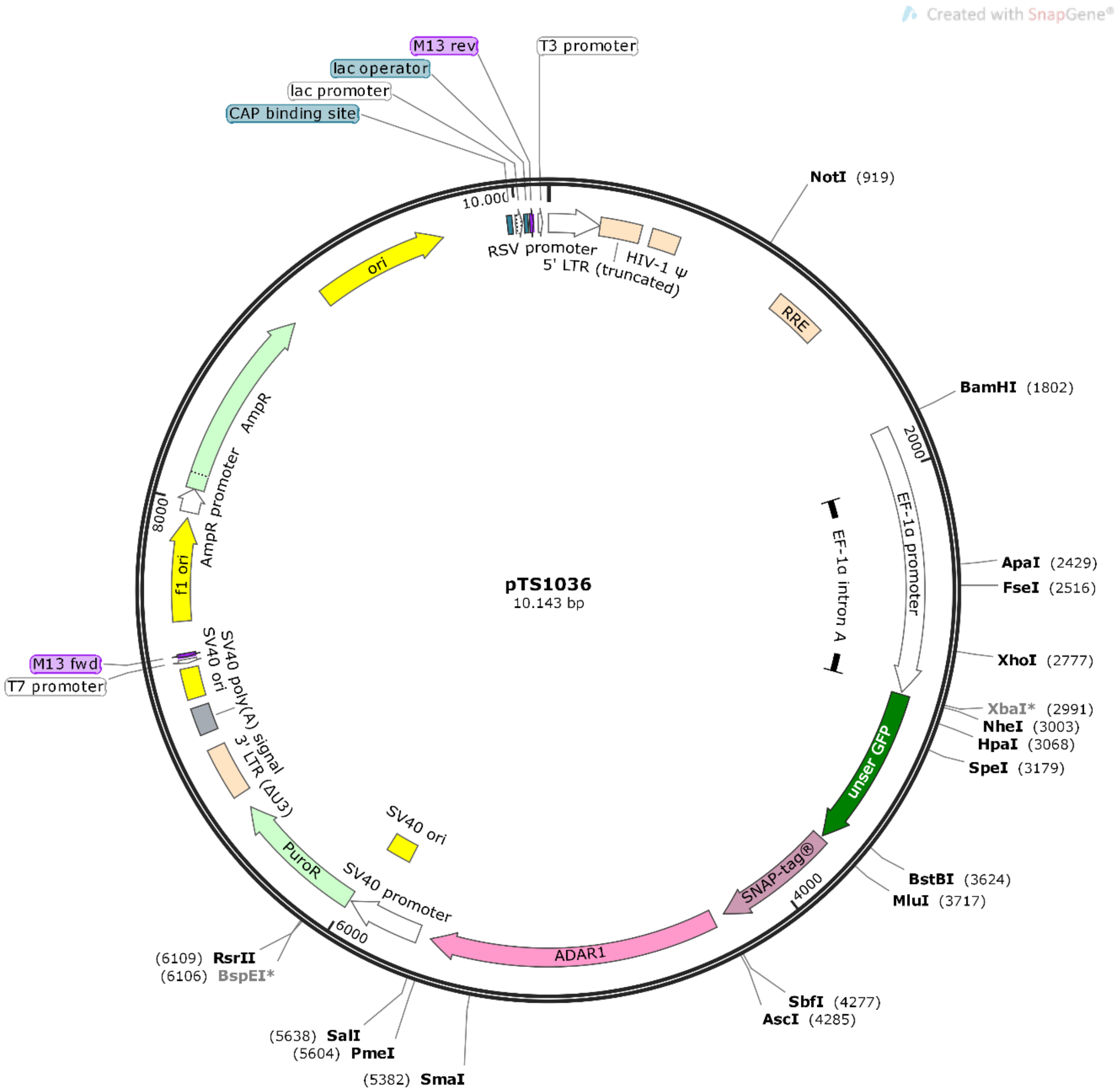

1 ATGTAGTCTT ATGCAATACT CTTGTAGTCT TGCAACATGG TAACGATGAG TTAGCAACAT
61 GCCTTACAAG GAGAGAAAAA GCACCGTGCA TGCCGATTGG TGGAAGTAAG GTGGTACGAT
121 CGTGCCTTAT TAGGAAGGCA ACAGACGGGT CTGACATGGA TTGGACGAAC CACTGAATTG
181 CCGCATTGCA GAGATATTGT ATTTAAGTGC CTAGCTCGAT ACATAAACGG GTCTCTCTGG
241 TTAGACCAGA TCTGAGCCTG GGAGCTCTCT GGCTAACTAG GGAACCCACT GCTTAAGCCT
301 CAATAAAGCT TGCCTTGAGT GCTTCAAGTA GTGTGTGCCC GTCTGTTGTG TGACTCTGGT
361 AACTAGAGAT CCCTCAGACC CTTTTAGTCA GTGTGGAAAA TCTCTAGCAG TGGCGCCCGA
421 ACAGGGACTT GAAAGCGAAA GGGAAACCAG AGGAGCTCTC TCGACGCAGG ACTCGGCTTG
481 CTGAAGCGCG CACGGCAAGA GGCGAGGGGC GGCGACTGGT GAGTACGCCA AAAATTTTGA
541 CTAGCGGAGG CTAGAAGGAG AGAGATGGGT GCGAGAGCGT CAGTATTAAG CGGGGGAGAA
601 TTAGATCGCG ATGGGAAAAA ATTCGGTTAA GGCCAGGGGG AAAGAAAAAA TATAAATTAA
661 AACATATAGT ATGGGCAAGC AGGGAGCTAG AACGATTCGC AGTTAATCCT GGCCTGTTAG
721 AAACATCAGA AGGCTGTAGA CAAATACTGG GACAGCTACA ACCATCCCTT CAGACAGGAT

781 CAGAAGAACT TAGATCATTA TATAATACAG TAGCAACCCT CTATTGTGTG CATCAAAGGA
841 TAGAGATAAA AGACACCAAG GAAGCTTTAG ACAAGATAGA GGAAGAGCAA AACAAAAGTA
901 AGACCACCGC ACAGCAAGCG GCCGCTGATC TTCAGACCTG GAGGAGGAGA TATGAGGGAC
961 AATTGGAGAA GTGAATTATA TAAATATAAA GTAGTAAAAA TTGAACCATT AGGAGTAGCA
1021 CCCACCAAGG CAAAGAGAAG AGTGGTGCAG AGAGAAAAAA GAGCAGTGGG AATAGGAGCT
1081 TTGTTCCTTG GGTTCTTGGG AGCAGCAGGA AGCACTATGG GCGCAGCGTC AATGACGCTG
1141 ACGGTACAGG CCAGACAATT ATTGTCTGGT ATAGTGCAGC AGCAGAACAA TTTGCTGAGG
1201 GCTATTGAGG CGCAACAGCA TCTGTTGCAA CTCACAGTCT GGGGCATCAA GCAGCTCCAG
1261 GCAAGAATCC TGGCTGTGGA AAGATACCTA AAGGATCAAC AGCTCCTGGG GATTTGGGGT
1321 TGCTCTGGAA AACTCATTTG CACCACTGCT GTGCCTTGGA ATGCTAGTTG GAGTAATAAA
1381 TCTCTGGAAC AGATTTGGAA TCACACGACC TGGATGGAGT GGGACAGAGA AATTAACAAT
1441 TACACAAGCT TAATACACTC CTTAATTGAA GAATCGCAAA ACCAGCAAGA AAAGAATGAA
1501 CAAGAATTAT TGGAATTAGA TAAATGGGCA AGTTTGTGGA ATTGGTTTAA CATAACAAAT
1561 TGGCTGTGGT ATATAAAATT ATTCATAATG ATAGTAGGAG GCTTGGTAGG TTTAAGAATA
1621 GTTTTTGCTG TACTTTCTAT AGTGAATAGA GTTAGGCAGG GATATTCACC ATTATCGTTT
1681 CAGACCCACC TCCCAACCCC GAGGGGACCC GACAGGCCCG AAGGAATAGA AGAAGAAGGT
1741 GGAGAGAGAG ACAGAGACAG ATCCATTCGA TTAGTGAACG GATCTCGACG GTATCGCTAG
1801 TGGATCCGTG AGGCTCCGGT GCCCGTCAGT GGGCAGAGCG CACATCGCCC ACAGTCCCCG
1861 AGAAGTTGGG GGGAGGGGTC GGCAATTGAA CCGGTGCCTA GAGAAGGTGG CGCGGGGTAA
1921 ACTGGGAAAG TGATGTCGTG TACTGGCTCC GCCTTTTTCC CGAGGGTGGG GGAGAACCGT
1981 ATATAAGTGC AGTAGTCGCC GTGAACGTTC TTTTTCGCAA CGGGTTTGCC GCCAGAACAC
2041 AGGTAAGTGC CGTGTGTGGT TCCCGCGGGC CTGGCCTCTT TACGGGTTAT GGCCCTTGCG
2101 TGCCTTGAAT TACTTCCACC TGGCTGCAGT ACGTGATTCT TGATCCCGAG CTTCGGGTTG
2161 GAAGTGGGTG GGAGAGTTCG AGGCCTTGCG CTTAAGGAGC CCCTTCGCCT CGTGCTTGAG
2221 TTGAGGCCTG GCCTGGGCGC TGGGGCCGCC GCGTGCGAAT CTGGTGGCAC CTTCGCGCCT
2281 GTCTCGCTGC TTTCGATAAG TCTCTAGCCA TTTAAAATTT TTGATGACCT GCTGCGACGC
2341 TTTTTTTCTG GCAAGATAGT CTTGTAAATG CGGGCCAAGA TCTGCACACT GGTATTTCGG
2401 TTTTTGGGGC CGCGGGCGGC GACGGGGCCC GTGCGTCCCA GCGCACATGT TCGGCGAGGC
2461 GGGGCCTGCG AGCGCGGCCA CCGAGAATCG GACGGGGGTA GTCTCAAGCT GGCCGGCCTG
2521 CTCTGGTGCC TGGCCTCGCG CCGCCGTGTA TCGCCCCGCC CTGGGCGGCA AGGCTGGCCC
2581 GGTCGGCACC AGTTGCGTGA GCGGAAAGAT GGCCGCTTCC CGGCCCTGCT GCAGGGAGCT
2641 CAAAATGGAG GACGCGGCGC TCGGGAGAGC GGGCGGGTGA GTCACCCACA CAAAGGAAAA
2701 GGGCCTTTCC GTCCTCAGCC GTCGCTTCAT GTGACTCCAC GGAGTACCGG GCGCCGTCCA
2761 GGCACCTCGA TTAGTTCTCG AGCTTTTGGA GTACGTCGTC TTTAGGTTGG GGGGAGGGGT
2821 TTTATGCGAT GGAGTTTCCC CACACTGAGT GGGTGGAGAC TGAAGTTAGG CCAGCTTGGC
2881 ACTTGATGTA ATTCTCCTTG GAATTTGCCC TTTTTGAGTT TGGATCTTGG TTCATTCTCA
2941 AGCCTCAGAC AGTGGTTCAA AGTTTTTTTC TTCCATTTCA GGTGTCGTGA TCTAGAACCA
3001 TGGCTAGCAA AGGAGAAGAA CTCTTCACTG GAGTTGTCCC AATTCTTGTT GAATTAGATG
3061 GTGATGTTAA CGGCCACAAG TTCTCTGTCA GTGGAGAGGG TGAAGGTGAT GCAACATACG
3121 GAAAACTTAC CCTGAAGTTC ATCTGCACTA CTGGCAAACT GCCTGTTCCG TGGCCGACAC
3181 TAGTGACGAC GCTCTGCTAT GGCGTCCAGT GCTTTTCAAG ATACCCGGAT CACATGAAAC
3241 GGCATGACTT TTTCAAGAGT GCCATGCCCG AAGGTTATGT ACAGGAAAGG ACCATCTTCT
3301 TCAAAGATGA CGGCAACTAC AAGACACGTG CTGAAGTCAA GTTTGAAGGT GATACCCTTG
3361 TTAATAGAAT CGAGTTAAAA GGTATTGACT TCAAGGAAGA TGGCAACATT CTGGGACACA
3421 AATTGGAATA CAACTATAAC TCACACAATG TATACATCAT GGCAGACAAA CAAAAGAATG
3481 GAATCAAAGT GAACTTCAAG ACCCGCCACA ACATTGAAGA TGGAAGCGTT CAACTAGCAG
3541 ACCATTATCA ACAAAATACT CCAATTGGCG ATGGCCCTGT CCTTTTACCA GACAACCATT
3601 ACCTGTCCAC ACAATCTGCC CTTTCGAAAG ATCCCAACGA AAAGAGAGAC CACATGGTCC
3661 TTCTTGAGTT TGTAACAGCT GCTGGGATTA CACATGGCAT GGATGAACTA TACAAAAACGC
3721 GTACCATGGA CAAAGACTGC GAAATGAAGC GCACCACCCT GGATAGCCCT CTGGGCAAGC
3781 TGGAACTGTC TGGGTGCGAA CAGGGCCTGC ACCGTATCAT CTTCCTGGGC AAAGGAACAT
3841 CTGCCGCCGA CGCCGTGGAA GTGCCTGCCC CAGCCGCCGT GCTGGGCGGA CCAGAGCCAC
3901 TGATGCAGGC CACCGCCTGG CTCAACGCCT ACTTTCACCA GCCTGAGGCC ATCGAGGAGT
3961 TCCCTGTGCC AGCCCTGCAC CACCCAGTGT TCCAGCAGGA GAGCTTTACC CGCCAGGTGC
4021 TGTGGAAACT GCTGAAAGTG GTGAAGTTCG GAGAGGTCAT CAGCTACAGC CACCTGGCCG
4081 CCCTGGCCGG CAATCCCGCC GCCACCGCCG CCGTGAAAAC CGCCCTGAGC GGAAATCCCG
4141 TGCCCATTCT GATCCCCTGC CACCGGGTGG TGCAGGGCGA CCTGGACGTG GGGGGCTACG
4201 AGGGCGGGCT CGCCGTGAAA GAGTGGCTGC TGGCCCACGA GGGCCACAGA CTGGGCAAGC
4261 CTGGGCTGGG TCCTGCAGGC GGAGGCGCGC CAGGGTCTGG CGGCGGCAGT AAGGCAGAAC
4321 GCATGGGTTT CACAGAGGTA ACCCCAGTGA CAGGGGCCAG TCTCAGAAGA ACTATGCTCC
4381 TCCTCTCAAG GTCCCCAGAA GCACAGCCAA AGACACTCCC TCTCACTGGC AGCACCTTCC

4441 ATGACCAGAT AGCCATGCTG AGCCACCGGT GCTTCAACAC TCTGACTAAC AGCTTCCAGC
4501 CCTCCTTGCT CGGCCGCAAG ATTCTGGCCG CCATCATTAT GAAAAAAGAC TCTGAGGACA
4561 TGGGTGTCGT CGTCAGCTTG GGAACAGGGA ATCGCTGTGT AAAAGGAGAT TCTCTCAGCC
4621 TAAAAGGAGA AACTGTCAAT GACTGCCATG CAGAAATAAT CTCCCGGAGA GGCTTCATCA
4681 GGTTTCTCTA CAGTGAGTTA ATGAAATACA ACTCCCAGAC TGCGAAGGAT AGTATATTTG
4741 AACCTGCTAA GGGAGGAGAA AAGCTCCAAA TAAAAAAGAC TGTGTCATTC CATCTGTATA
4801 TCAGCACTGC TCCGTGTGGA GATGGCGCCC TCTTTGACAA GTCCTGCAGC GACCGTGCTA
4861 TGGAAAGCAC AGAATCCCGC CACTACCCTG TCTTCGAGAA TCCCAAACAA GGAAAGCTCC
4921 GCACCAAGGT GGAGAACGGA GAAGGCACAA TCCCTGTGGA ATCCAGTGAC ATTGTGCCTA
4981 CGTGGGATGG CATTCGGCTC GGGGAGAGAC TCCGTACCAT GTCCTGTAGT GACAAAATCC
5041 TACGCTGGAA CGTGCTGGGC CTGCAAGGGG CACTGTTGAC CCACTTCCTG CAGCCCATTT
5101 ATCTCAAATC TGTCACATTG GGTTACCTTT TCAGCCAAGG GCATCTGACC CGTGCTATTT
5161 GCTGTCGTGT GACAAGAGAT GGGAGTGCAT TTGAGGATGG ACTACGACAT CCCTTTATTG
5221 TCAACCACCC CAAGGTTGGC AGAGTCAGCA TATATGATTC CAAAAGGCAA TCCGGGAAGA
5281 CTAAGGAGAC AAGCGTCAAC TGGTGTCTGG CTGATGGCTA TGACCTGGAG ATCCTGGACG
5341 GTACCAGAGG CACTGTGGAT GGGCCACGGA ATGAATTGTC CCGGGTCTCC AAAAAGAACA
5401 TTTTTCTTCT ATTTAAGAAG CTCTGCTCCT TCCGTTACCG CAGGGATCTA CTGAGACTCT
5461 CCTATGGTGA GGCCAAGAAA GCTGCCCGTG ACTACGAGAC GGCCAAGAAC TACTTCAAAA
5521 AAGGCCTGAA GGATATGGGC TATGGGAACT GGATTAGCAA ACCCCAGGAG GAAAAGAACT
5581 TTTATCTCTG CCCAGTATAA GTTTAAACCT GCTGATCAGC CTCGACTGTG CCTTCTAGTC
5641 GACCCTGTGG AATGTGTGTC AGTTAGGGTG TGGAAAGTCC CCAGGCTCCC CAGCAGGCAG
5701 AAGTATGCAA AGCATGCATC TCAATTAGTC AGCAACCAGG TGTGGAAAGT CCCCAGGCTC
5761 CCCAGCAGGC AGAAGTATGC AAAGCATGCA TCTCAATTAG TCAGCAACCA TAGTCCCGCC
5821 CCTAACTCCG CCCATCCCGC CCCTAACTCC GCCCAGTTCC GCCCATTCTC CGCCCCATGG
5881 CTGACTAATT TTTTTTATTT ATGCAGAGGC CGAGGCCGCC TCGGCCTCTG AGCTATTCCA
5941 GAAGTAGTGA GGAGGCTTTT TTGGAGGCCT AGGCTTTTGC AAAAAGCTTA CCATGACCGA
6001 GTACAAGCCC ACGGTGCGCC TCGCCACCCG CGACGACGTC CCCAGGGCCG TACGCACCCT
6061 CGCCGCCGCG TTCGCCGACT ACCCCGCCAC GCGCCACACC GTCGATCCGG ACCGCCACAT
6121 CGAGCGGGTC ACCGAGCTGC AAGAACTCTT CCTCACGCGC GTCGGGCTCG ACATCGGCAA
6181 GGTGTGGGTC GCGGACGACG GCGCCGCGGT GGCGGTCTGG ACCACGCCGG AGAGCGTCGA
6241 AGCGGGGGCG GTGTTCGCCG AGATCGGCCC GCGCATGGCC GAGTTGAGCG GTTCCCGGCT
6301 GGCCGCGCAG CAACAGATGG AAGGCCTCCT GGCGCCGCAC CGGCCCAAGG AGCCCGCGTG
6361 GTTCCTGGCC ACCGTCGGCG TCTCGCCCGA CCACCAGGGC AAGGGTCTGG GCAGCGCCGT
6421 CGTGCTCCCC GGAGTGGAGG CGGCCGAGCG CGCCGGGGTG CCCGCCTTCC TGGAGACCTC
6481 CGCGCCCCGC AACCTCCCCT TCTACGAGCG GCTCGGCTTC ACCGTCACCG CCGACGTCGA
6541 GTGCCCGAAG GACCGCGCGA CCTGGTGCAT GACCCGCAAG CCCGGTGCCT GAGGTACCTT
6601 TAAGACCAAT GACTTACAAG GCAGCTGTAG ATCTTAGCCA CTTTTTAAAA GAAAAGGGGG
6661 GACTGGAAGG GCTAATTCAC TCCCAACGAA GACAAGATCT GCTTTTTGCT TGTACTGGGT
6721 CTCTCTGGTT AGACCAGATC TGAGCCTGGG AGCTCTCTGG CTAACTAGGG AACCCACTGC
6781 TTAAGCCTCA ATAAAGCTTG CCTTGAGTGC TTCAAGTAGT GTGTGCCCGT CTGTTGTGTG
6841 ACTCTGGTAA CTAGAGATCC CTCAGACCCT TTTAGTCAGT GTGGAAAATC TCTAGCAGTA
6901 GTAGTTCATG TCATCTTATT ATTCAGTATT TATAACTTGC AAAGAAATGA ATATCAGAGA
6961 GTGAGAGGAA CTTGTTTATT GCAGCTTATA ATGGTTACAA ATAAAGCAAT AGCATCACAA
7021 ATTTCACAAA TAAAGCATTT TTTTCACTGC ATTCTAGTTG TGGTTTGTCC AAACTCATCA
7081 ATGTATCTTA TCATGTCTGG CTCTAGCTAT CCCGCCCCTA ACTCCGCCCA TCCCGCCCCT
7141 AACTCCGCCC AGTTCCGCCC ATTCTCCGCC CCATGGCTGA CTAATTTTTT TTATTTATGC
7201 AGAGGCCGAG GCCGCCTCGG CCTCTGAGCT ATTCCAGAAG TAGTGAGGAG GCTTTTTTGG
7261 AGGCCTAGGG ACGTACCCAA TTCGCCCTAT AGTGAGTCGT ATTACGCGCG CTCACTGGCC
7321 GTCGTTTTAC AACGTCGTGA CTGGGAAAAC CCTGGCGTTA CCCAACTTAA TCGCCTTGCA
7381 GCACATCCCC CTTTCGCCAG CTGGCGTAAT AGCGAAGAGG CCCGCACCGA TCGCCCTTCC
7441 CAACAGTTGC GCAGCCTGAA TGGCGAATGG GACGCGCCCT GTAGCGGCGC ATTAAGCGCG
7501 GCGGGTGTGG TGGTTACGCG CAGCGTGACC GCTACACTTG CCAGCGCCCT AGCGCCCGCT
7561 CCTTTCGCTT TCTTCCCTTC CTTTCTCGCC ACGTTCGCCG GCTTTCCCCG TCAAGCTCTA
7621 AATCGGGGGC TCCCTTTAGG GTTCCGATTT AGTGCTTTAC GGCACCTCGA CCCCAAAAAA
7681 CTTGATTAGG GTGATGGTTC ACGTAGTGGG CCATCGCCCT GATAGACGGT TTTTCGCCCT
7741 TTGACGTTGG AGTCCACGTT CTTTAATAGT GGACTCTTGT TCCAAACTGG AACAACACTC
7801 AACCCTATCT CGGTCTATTC TTTTGATTTA TAAGGGATTT TGCCGATTTC GGCCTATTGG
7861 TTAAAAAATG AGCTGATTTA ACAAAAATTT AACGCGAATT TTAACAAAAT ATTAACGCTT
7921 ACAATTTAGG TGGCACTTTT CGGGGAAATG TGCGCGGAAC CCCTATTTGT TTATTTTTCT
7981 AAATACATTC AAATATGTAT CCGCTCATGA GACAATAACC CTGATAAATG CTTCAATAAT
8041 ATTGAAAAAG GAAGAGTATG AGTATTCAAC ATTTCCGTGT CGCCCTTATT CCCTTTTTTG

8101 CGGCATTTTG CCTTCCTGTT TTTGCTCACC CAGAAACGCT GGTGAAAGTA AAAGATGCTG
8161 AAGATCAGTT GGGTGCACGA GTGGGTTACA TCGAACTGGA TCTCAACAGC GGTAAGATCC
8221 TTGAGAGTTT TCGCCCCGAA GAACGTTTTC CAATGATGAG CACTTTTAAA GTTCTGCTAT
8281 GTGGCGCGGT ATTATCCCGT ATTGACGCCG GGCAAGAGCA ACTCGGTCGC CGCATACACT
8341 ATTCTCAGAA TGACTTGGTT GAGTACTCAC CAGTCACAGA AAAGCATCTT ACGGATGGCA
8401 TGACAGTAAG AGAATTATGC AGTGCTGCCA TAACCATGAG TGATAACACT GCGGCCAACT
8461 TACTTCTGAC AACGATCGGA GGACCGAAGG AGCTAACCGC TTTTTTGCAC AACATGGGGG
8521 ATCATGTAAC TCGCCTTGAT CGTTGGGAAC CGGAGCTGAA TGAAGCCATA CCAAACGACG
8581 AGCGTGACAC CACGATGCCT GTAGCAATGG CAACAACGTT GCGCAAACTA TTAACTGGCG
8641 AACTACTTAC TCTAGCTTCC CGGCAACAAT TAATAGACTG GATGGAGGCG GATAAAGTTG
8701 CAGGACCACT TCTGCGCTCG GCCCTTCCGG CTGGCTGGTT TATTGCTGAT AAATCTGGAG
8761 CCGGTGAGCG TGGGTCTCGC GGTATCATTG CAGCACTGGG GCCAGATGGT AAGCCCTCCC
8821 GTATCGTAGT TATCTACACG ACGGGGAGTC AGGCAACTAT GGATGAACGA AATAGACAGA
8881 TCGCTGAGAT AGGTGCCTCA CTGATTAAGC ATTGGTAACT GTCAGACCAA GTTTACTCAT
8941 ATATACTTTA GATTGATTTA AAACTTCATT TTTAATTTAA AAGGATCTAG GTGAAGATCC
9001 TTTTTGATAA TCTCATGACC AAAATCCCTT AACGTGAGTT TTCGTTCCAC TGAGCGTCAG
9061 ACCCCGTAGA AAAGATCAAA GGATCTTCTT GAGATCCTTT TTTTCTGCGC GTAATCTGCT
9121 GCTTGCAAAC AAAAAAACCA CCGCTACCAG CGGTGGTTTG TTTGCCGGAT CAAGAGCTAC
9181 CAACTCTTTT TCCGAAGGTA ACTGGCTTCA GCAGAGCGCA GATACCAAAT ACTGTTCTTC
9241 TAGTGTAGCC GTAGTTAGGC CACCACTTCA AGAACTCTGT AGCACCGCCT ACATACCTCG
9301 CTCTGCTAAT CCTGTTACCA GTGGCTGCTG CCAGTGGCGA TAAGTCGTGT CTTACCGGGT
9361 TGGACTCAAG ACGATAGTTA CCGGATAAGG CGCAGCGGTC GGGCTGAACG GGGGGTTCGT
9421 GCACACAGCC CAGCTTGGAG CGAACGACCT ACACCGAACT GAGATACCTA CAGCGTGAGC
9481 TATGAGAAAG CGCCACGCTT CCCGAAGGGA GAAAGGCGGA CAGGTATCCG GTAAGCGGCA
9541 GGGTCGGAAC AGGAGAGCGC ACGAGGGAGC TTCCAGGGGG AAACGCCTGG TATCTTTATA
9601 GTCCTGTCGG GTTTCGCCAC CTCTGACTTG AGCGTCGATT TTTGTGATGC TCGTCAGGGG
9661 GGCGGAGCCT ATGGAAAAAC GCCAGCAACG CGGCCTTTTT ACGGTTCCTG GCCTTTTGCT
9721 GGCCTTTTGC TCACATGTTC TTTCCTGCGT TATCCCCTGA TTCTGTGGAT AACCGTATTA
9781 CCGCCTTTGA GTGAGCTGAT ACCGCTCGCC GCAGCCGAAC GACCGAGCGC AGCGAGTCAG
9841 TGAGCGAGGA AGCGGAAGAG CGCCCAATAC GCAAACCGCC TCTCCCCGCG CGTTGGCCGA
9901 TTCATTAATG CAGCTGGCAC GACAGGTTTC CCGACTGGAA AGCGGGCAGT GAGCGCAACG
9961 CAATTAATGT GAGTTAGCTC ACTCATTAGG CACCCCAGGC TTTACACTTT ATGCTTCCGG
10021 CTCGTATGTT GTGTGGAATT GTGAGCGGAT AACAATTTCA CACAGGAAAC AGCTATGACC
10081 ATGATTACGC CAAGCGCGCA ATTAACCCTC ACTAAAGGGA ACAAAAGCTG GAGCTGCAAG
10141 CTT
