# Supplementary material for: An improved SNAP-ADAR tool enables efficient RNA base editing to interfere with post-translational protein modification
Source: Nat Commun. 2024 Aug 5;15:6615. doi: 10.1038/s41467-024-50395-w (PMC11300799; doi:10.1038/s41467-024-50395-w)
Supplement: Supplementary file 3 — Description of Additional Supplementary Files [file 41467_2024_50395_MOESM3_ESM.pdf]

Description of Additional Supplementary Files

Supplementary Data 1. **List of target sites and guide RNA sequences**. Given are the unique number of each guide RNA, the target gene ID, the target site, target codon, guide RNA chemical modification pattern, the PTM modification type, the expected effect upon editing, the uniprot ID and a literature reference. gRNA sequence: [2´-O-methyl rNT], fN in blue = 2´-F-rNT, (RNA), {LNA}, I in orange = deoxy Inosine, *=Phosphorothioate. PTMi modification type: pY/S/T (phosphor tyrosine/serine/threonine), Ac (acetylation), Ub (ubitinylation), di-Me (diemethylation), GOF (gain of function mutation). Expected outcome upon editing: red – downregulation; green – upregulation.

Supplementary Data 2. List of qPCR primers.

Supplementary Data 3. List of sense DNA oligonucleotides to bind guide RNA. DNA sequences are given from 5´ to 3´.
