# Supplementary material for: An improved SNAP-ADAR tool enables efficient RNA base editing to interfere with post-translational protein modification
Source: Nat Commun. 2024 Aug 5;15:6615. doi: 10.1038/s41467-024-50395-w (PMC11300799; doi:10.1038/s41467-024-50395-w)
Supplement: Supplementary file 5 — Reporting summary [file 41467_2024_50395_MOESM5_ESM.pdf]

nature portfolio

Corresponding author(s): Thorsten Stafforst

Last updated by author(s): 2024/06/07

# Reporting Summary

Nature Portfolio wishes to improve the reproducibility of the work that we publish. This form provides structure for consistency and transparency in reporting. For further information on Nature Portfolio policies, see our Editorial Policies and the Editorial Policy Checklist.

## Statistics

For all statistical analyses, confirm that the following items are present in the figure legend, table legend, main text, or Methods section.

| n/a | Confirmed | |
|---|---|---|
| ☐ | ☒ | The exact sample size (*n*) for each experimental group/condition, given as a discrete number and unit of measurement |
| ☐ | ☒ | A statement on whether measurements were taken from distinct samples or whether the same sample was measured repeatedly |
| ☐ | ☒ | The statistical test(s) used AND whether they are one- or two-sided<br>*Only common tests should be described solely by name; describe more complex techniques in the Methods section.* |
| ☒ | ☐ | A description of all covariates tested |
| ☒ | ☐ | A description of any assumptions or corrections, such as tests of normality and adjustment for multiple comparisons |
| ☐ | ☒ | A full description of the statistical parameters including central tendency (e.g. means) or other basic estimates (e.g. regression coefficient) AND variation (e.g. standard deviation) or associated estimates of uncertainty (e.g. confidence intervals) |
| ☐ | ☒ | For null hypothesis testing, the test statistic (e.g. *F*, *t*, *r*) with confidence intervals, effect sizes, degrees of freedom and *P* value noted<br>*Give P values as exact values whenever suitable.* |
| ☒ | ☐ | For Bayesian analysis, information on the choice of priors and Markov chain Monte Carlo settings |
| ☒ | ☐ | For hierarchical and complex designs, identification of the appropriate level for tests and full reporting of outcomes |
| ☒ | ☐ | Estimates of effect sizes (e.g. Cohen's *d*, Pearson's *r*), indicating how they were calculated |

*Our web collection on statistics for biologists contains articles on many of the points above.*

## Software and code

Policy information about availability of computer code

| | |
|---|---|
| Data collection | No custom-made code was required to collect data. |
| Data analysis | Data were analyzed using Excel 2016 and GraphPad Prism 8,<br>Figures were created with CorelDraw 2017 and BioRender.com,<br>Manuscript was written with Word 2016,<br>qPCR analysis was performed using the 7500 data analysis software v2.3.<br>Heatmap was made using Jupyter Notebook (6.4.12).<br>NGS data was analysed using STAR (v. 2.4.2a) to align RNA-seq reads to the hg19 reference genome and ran RSEM (v. 1.2.21) on the alignments to calculate read counts and TPM values. Using read counts, gene expression was analyzed for all genes with TPM ≥ 2 (for both replicates) with the R package DESeq2.<br>Microscopy images were acquired and deconvoluted using NIS element software (version 14.0.0.0) and pseudo colored using ImageJ (1.54f)<br>Colocalization analysis was made using JACoP plugin in ImageJ (1.54f)<br>All the grey value measurements for western blots was done using ImageJ (1.53q).<br>Guide RNA hairpin folding secondary structure calculated with NUPACK online tool.<br>Oligo Calc: Oligonucleotide Properties Calculator, online tool was utilized for calculating melting temperatures of primers.<br>Sanger sequence traces were analyzed using SNAP-Gene (version 4.2.11), |

For manuscripts utilizing custom algorithms or software that are central to the research but not yet described in published literature, software must be made available to editors and reviewers. We strongly encourage code deposition in a community repository (e.g. GitHub). See the Nature Portfolio guidelines for submitting code & software for further information.

## Data

Policy information about availability of data

All manuscripts must include a data availability statement. This statement should provide the following information, where applicable:

- Accession codes, unique identifiers, or web links for publicly available datasets
- A description of any restrictions on data availability
- For clinical datasets or third party data, please ensure that the statement adheres to our policy

The raw DESeq data is available via the NCBI GEO server under the project number GSE264114.

## Research involving human participants, their data, or biological material

Policy information about studies with human participants or human data. See also policy information about sex, gender (identity/presentation), and sexual orientation and race, ethnicity and racism.

| | |
|---|---|
| Reporting on sex and gender | No research involving human participants. |
| Reporting on race, ethnicity, or other socially relevant groupings | No research involving human participants. |
| Population characteristics | No research involving human participants. |
| Recruitment | No research involving human participants. |
| Ethics oversight | No research involving human participants. |

Note that full information on the approval of the study protocol must also be provided in the manuscript.

# Field-specific reporting

Please select the one below that is the best fit for your research. If you are not sure, read the appropriate sections before making your selection.

☒ Life sciences ☐ Behavioural & social sciences ☐ Ecological, evolutionary & environmental sciences

For a reference copy of the document with all sections, see nature.com/documents/nr-reporting-summary-flat.pdf

# Life sciences study design

All studies must disclose on these points even when the disclosure is negative.

| | |
|---|---|
| Sample size | The group sizes for cell culture experiments were selected based on the prior knowledge of variation. Experiments for evaluating editing yields via Sanger sequencing were mostly done in N=3 biological replicates in rare cases in N=4 or N=2. Everywhere (in bar diagrams or box plots) data points are displayed individually.<br><br>In case of heat maps the mean ± s.d. of N = 2 was displayed.<br><br>The evaluation of the fold change via the qPCR assay for cell culture experiments was done with N=2 biological replicates.<br>For Western blot analysis, N = 2 or N=3 biological replicated were determined, for N = 3 t-test statisitcs were analyzed. |
| Data exclusions | No data was excluded. |
| Replication | All experiments could be reproduced, as shown in the manuscript, the number of replications and nature of replicates is always given in the figure caption. Everywhere (in bar diagrams or box plots) data points are displayed individually. |
| Randomization | All samples were treated according to the same protocols side-by-side with the respective controls and thus, there was no requirement for randomization. |
| Blinding | No Blinding was performed. |

# Reporting for specific materials, systems and methods

We require information from authors about some types of materials, experimental systems and methods used in many studies. Here, indicate whether each material, system or method listed is relevant to your study. If you are not sure if a list item applies to your research, read the appropriate section before selecting a response.

## Materials & experimental systems

| n/a | Involved in the study |
|---|---|
| ☐ | ☒ Antibodies |
| ☐ | ☒ Eukaryotic cell lines |
| ☒ | ☐ Palaeontology and archaeology |
| ☒ | ☐ Animals and other organisms |
| ☒ | ☐ Clinical data |
| ☒ | ☐ Dual use research of concern |
| ☒ | ☐ Plants |

## Methods

| n/a | Involved in the study |
|---|---|
| ☒ | ☐ ChIP-seq |
| ☒ | ☐ Flow cytometry |
| ☒ | ☐ MRI-based neuroimaging |

# Antibodies

Antibodies used

Rabbit α-SNAP-tag -P9310S, New England Biolabs
α-SNAP Tag Polyclonal Antibody-CAB4255, Thermo-Fisher Scientific
Mouse-α-GAPDH Loading Control Monoclonal Antibody (GA1R)-MA5-15738, Thermo-Fisher Scientific
Mouse-α-GAPDH Monoclonal Antibody (6C5)-AM4300, Thermo-Fisher Scientific
Rabbit-α-Gapdh (D16H11) XP  5174T/5174S, Cell Signaling
Mouse α-ACTB (A5441), Sigma Aldrich
pSTAT1(Y701) 9167, Cell Signaling
STAT1 (42H3) Rabbit 9175, Cell Signaling
STAT1 mouse 9176, Cell Signaling
Goat α-Rabbit Alexa Fluor 594 8889S, Cell Signaling
Goat α-Mouse HRP (new) 115-035-003, Jackson Immuno Research Laboratories
Goat α-Rabbit HRP (new) 111-035-003, Jackson Immuno Research Laboratories
STAT2 rabbit 72604, Cell Signaling
IFNGR1 rabbit 34808, Cell Signaling
JAK1 mouse 50996, Cell Signaling
IFNAR1 rabbit MA5-32006, Thermo-Fisher Scientific
STAT1 Rabbit 9172, Cell Signaling

Validation

Rabbit α-SNAP (New England Bioloabs, P9310S), validated in our laboratory via overexpression of SNAP-tag fusion proteins.
α-SNAP Tag Polyclonal Antibody-CAB4255, Thermo-Fisher Scientific see PMID: 30543635
Mouse α-GAPDH (Thermo Scientific, GA1R) see PMID: 31519936
Mouse-α-GAPDH Monoclonal Antibody (6C5)-AM4300, Thermo-Fisher Scientific see PMID: 35879364
Rabbit-α-Gapdh (D16H11) XP  5174T/5174S, Cell Signaling see PMID: 36309506
Mouse α-ACTB (Sigma Aldrich,A5441) see PMID: 30814728
pSTAT1(Y701) 9167, Cell Signaling see PMID: 35484149
STAT1 (42H3) Rabbit 9175, Cell Signaling see PMID: 31043744
STAT1 mouse 9176, Cell Signaling see PMID: 35013300
Goat α-Rabbit Alexa Fluor 594 8889S, Cell Signaling see PMID: 32323797
STAT2 rabbit 72604, Cell Signaling see PMID: 32778820
IFNGR1 rabbit 34808, Cell Signaling see PMID: 35127252
JAK1 mouse 50996, Cell Signaling see PMID: 37925520
IFNAR1 rabbit MA5-32006, Thermo-Fisher Scientific see PMID: 33615517
STAT1 Rabbit 9172, Cell Signaling see PMID: 36755096

# Eukaryotic cell lines

Policy information about cell lines and Sex and Gender in Research

Cell line source(s)

HeLa (ACC 57, DSMZ, Braunschweig, Germany)
A549 (ACC 107, DSMZ, Braunschweig, Germany)
Ad293 cells (Cat. No. 240085, Agilent Technologies)
HEK-Flp-In T-Rex-A1p110 (cat. no. R78007, Thermo Fisher scientific, stably transfected with ADAR1 p110 vector in our lab)
HEK 293FT-cells (Cat. No. R70007, ThermoFisher Scientific)
Primary cells, NHA (Normal Human Astrocytes, Cat.no. CC-2565, LONZA)
Primary cells, H-RPE (Human Retinal Pigment Epithelial Cells, Cat. no. 00194987, LONZA)

Authentication

Authentication via STR profiling was performed by the commercial suppliers before purchase of the material. Cell lines were not additionally authenticated by us.

Mycoplasma contamination

Ad293, NHA and RPE were certified as mycoplasma-free by the commercial suppliers. HeLa, A549, Flp-In T-Rex-A1p110 , HEK 293FT have been tested as mycoplasma-free in house.

Commonly misidentified lines
(See ICLAC register)

None were used.

## Plants

| | |
|---|---|
| Seed stocks | No plants were used for this study. |
| Novel plant genotypes | No plants were used for this study. |
| Authentication | No plants were used for this study. |
